# Dual-mode screening system for rapid and accurate aflatoxin B1 detection in edible oils

**DOI:** 10.1016/j.fochx.2026.104325

**Published:** 2026-08-17

**Authors:** Guangyao Ying, Tingting Wang, Kunlun Li, Zheng Lu, Yuxin Wang, Gangjian Lin, Jun Li, Huili Xia, Liang Hong

**Affiliations:** a Taizhou Institute for Food and Drug Control, Taizhou 318000, China; b Taizhou Key Laboratory of Model Animal and Preclinical Pharmaceutical Research, Taizhou 318000, China; c Key Laboratory for Quality Safety and Quality Improvement of Characteristic Agricultural Products in Taizhou City, Taizhou 318000, China; d Taizhou Center for Disease Control and Prevention, Taizhou 318000, China; e School of Pharmaceutical Sciences, Jinhua Academy, Zhejiang Chinese Medical University, Hangzhou 310053, China

**Keywords:** Portable flow cytometer, Liquid chromatography-tandem mass spectrometry, Edible oil, Aflatoxin B1, Rapid screening

## Abstract

This study established a dual-mode system integrating a portable flow cytometer with UHPLC-QqQ MS/MS for rapid AFB1 detection in edible oils. A competitive immunofluorescence sensor used streptavidin-magnetic microspheres with biotin-AFB1-BSA, AFB1 antibody, and APC-labeled secondary antibody. The method showed a linear range of 0.025–25 ng/g (R^2^ = 0.9966), with IC10 of 0.26 ng/g and IC50 of 0.76 ng/g. Recoveries were 90.51–102.73% with precisions ≤9.54%. UHPLC-QqQ MS/MS achieved LODs of 0.20–0.40 ng/g and LOQs of 0.50–1.25 ng/g for AFB1, AFB2, AFG1, and AFG2, with recoveries of 88.99–107.12% and precisions of 1.09–10.58%. Validation with 29 commercial oils and 10 contaminated peanut batches found no AFB1 in market samples and 1.07–9.36 ng/g in contaminated ones, consistent with UHPLC-QqQ MS/MS. The system enables on-site high-throughput screening with a flow cytometric reading speed of approximately 1 min per sample and a batch throughput of ∼32 samples/h (96-well plate format), while maintaining laboratory-grade accuracy. By enabling efficient triage of large sample volumes at the point of sampling, this strategy provides a practical and cost-effective framework for strengthening aflatoxin surveillance in the edible oil supply chain.

## 1 Introduction

Aflatoxins are a group of highly toxic and carcinogenic mycotoxins. They are primarily produced by the filamentous fungi *Aspergillus flavus* and *Aspergillus parasiticus*, which can contaminate a wide range of agricultural commodities such as oilseeds (e.g., peanuts, soybeans) and their derived products like edible oils, especially under conditions of high temperature and humidity (Ting, Chang, Szonyi, and Gizachew (2020); Ma, Zhang, Sun, and Qi (2015)). Among them, aflatoxin B1 (AFB1) exhibits the highest toxicity and has been classified as a Group 1 carcinogen (carcinogenic to humans) by the International Agency for Research on Cancer (IARC) (Ostry, Malir, Toman, and Grosse (2017)). While *Aspergillus flavus* primarily produces AFB1 and AFB2, *Aspergillus parasiticus* typically produces AFB1, AFB2, AFG1, and AFG2. AFB1 is the most prevalent and potent carcinogen found in contaminated edible oils and oilseeds, and it may migrate into the oil during processing (Javanmardi et al. (2022)). Therefore, most countries and organizations have established strict maximum limits for aflatoxins in edible oils. For instance, the EU document COMMISSION REGULATION (EU) 2023/915 specifies a maximum level of 4 μg/kg for the sum of aflatoxins (B1, B2, G1, G2) in vegetable oils, with an individual limit of 2 μg/kg for AFB1 (European Commission (2023)), a stringent threshold established based on its potent hepatocarcinogenicity and extensive toxicological risk assessments.

Currently, the analytical methods for aflatoxins in edible oils can be broadly categorized into two groups: laboratory-based confirmatory methods, such as high-performance liquid chromatography with fluorescence detection (HPLC-FLD) and liquid chromatography-tandem mass spectrometry (LC-MS/MS); and on-site rapid screening techniques, such as lateral flow immunoassay (LFIA) strips and enzyme-linked immunosorbent assay (ELISA). However, a significant gap remains for techniques suitable for large-scale regulatory screening. The instrumental methods involve complex sample pretreatment and require sophisticated laboratory settings, making them impractical for rapid on-site deployment. Conversely, while LFIA is simple and fast, it typically provides only qualitative or semi-quantitative results with limited throughput (Kim, Lim, and Mo (2015)). ELISA offers better quantification but involves multiple liquid handling steps and longer incubation times (often >1 h), limiting its suitability for true on-site, high-throughput testing. Therefore, there is a pressing need for a detection strategy that combines quantitative capability, high throughput, and field portability to efficiently triage a large number of samples in the surveillance chain.

Flow cytometry, particularly with the recent development of compact benchtop instruments such as the Beckman Coulter CytoFLEX and BD Accuri C6 Plus (weighing <15 kg and transportable by a single operator), has emerged as a promising platform for on-site analysis. The microsphere-based flow cytometry (MFC) format offers high sensitivity, rapid analysis, and multiplexing potential for mycotoxin detection (Wang et al. (2013)); Wang et al. (2013); Zhang et al. (2017); Liu et al. (2018)). However, most existing MFC-based assays have relied on conventional large benchtop instruments under controlled laboratory conditions, involving sophisticated sample handling and standardized operational workflows. To date, no studies have reported the application of compact flow cytometry for the rapid field screening of aflatoxins in edible oils, representing a critical gap that requires methodological development and field validation.

To the best of our knowledge, this study represents the first reported application of a compact benchtop flow cytometer for the on-site screening of aflatoxins in edible oils. The scientific novelty of this work lies in three key aspects. First, unlike previous MFC-based mycotoxin assays that relied on conventional large-scale flow cytometers in controlled laboratory environments, the present study demonstrates that a portable instrument (CytoFLEX) can achieve comparable analytical sensitivity for aflatoxin detection directly in complex oil matrices after a simplified sample preparation. Second, rather than presenting MFC as a standalone assay, this work strategically integrates it with UHPLC-QqQ MS/MS into a coherent dual-mode workflow, in which MFC serves as a high-throughput, field-deployable first-tier screen (∼32 samples/h) and UHPLC-QqQ MS/MS provides definitive multi-analyte confirmation only for presumptive positive samples, thereby addressing the longstanding disconnect between rapid screening capability and regulatory-grade confirmatory accuracy. Third, a systematic optimization of the MFC immunoassay specific to the edible oil matrix was conducted, including the identification of PSA as a critical cleanup sorbent and the evaluation of antibody tolerance to co-existing organic solvents, which are practical considerations largely overlooked in earlier MFC studies focused on aqueous-based matrices. Collectively, these contributions bridge the gap between laboratory-based mycotoxin analysis and field-deployable surveillance, providing a practically validated and regulatory-relevant detection strategy for edible oil safety monitoring.

Therefore, the aim of this study was to develop and validate an integrated analytical strategy that combines rapid on-site screening with definitive laboratory confirmation for the efficient surveillance of aflatoxins in edible oils.

## 2 Materials and methods

### 2.1 Materials and reagents

Edible oil samples (peanut oil, rapeseed oil, soybean blended oil, rice bran oil, olive oil, walnut oil, sunflower seed oil, rice oil, linseed oil, corn oil, sesame oil, camellia oil) were purchased from two distinct grain markets and one large chain supermarket.

AFB1 polyclonal antibody (2.5 mg/mL) and biotin-modified AFB1 antigen (1 mg/mL) were obtained from Shandong Lvdu Biotechnology Co., Ltd. Streptavidin-modified magnetic microspheres (2.72 × 10^7^ particles/mL) were sourced from Weido Biotechnology Co., Ltd. (Suzhou). Phosphate-Buffered Saline(PBS), Phosphate Buffered Saline with Tween-20(PBST), 5% BSA blocking buffer, and APC-labeled fluorescent secondary antibody (2 mg/mL) were purchased from Beijing Solarbio Science & Technology Co., Ltd. Purified water was supplied by Hangzhou Wahaha Group Co., Ltd. Methanol and acetonitrile (HPLC grade) were acquired from Sigma-Aldrich (Shanghai) Trading Co., Ltd. ProClin300 preservative was provided by Beyotime Biotechnology (Shanghai). Mycotoxin standards, including aflatoxins (AFB1, AFB2, AFG1, AFG2), ochratoxin A (OTA), deoxynivalenol (DON), zearalenone (ZEN), patulin (PAT), and fumonisins (FB1, FB2), were purchased from Pribolab Bioengineering Co., Ltd. (Qingdao). Analytical quality control sample for aflatoxins B and G in peanut oil, were purchased from TanMo Reference Materials Company Ltd.

### 2.2 Instruments

CytoFLEX flow cytometer (Beckman Coulter); VORTEX 3 vortex mixer and MATRIX Orbital Delta Plus thermomixer (IKA (Guangzhou) Instrument Equipment Co., Ltd.); BeyoMag magnetic separation rack (Beyotime Biotechnology); Multi Reax multi-tube vortex mixer (Heidolph Instruments (Shanghai) Co., Ltd.); 5500+ UHPLC-QqQ MS/MS (AB SCIEX).

### 2.3 Methods

**Fig. 1:**
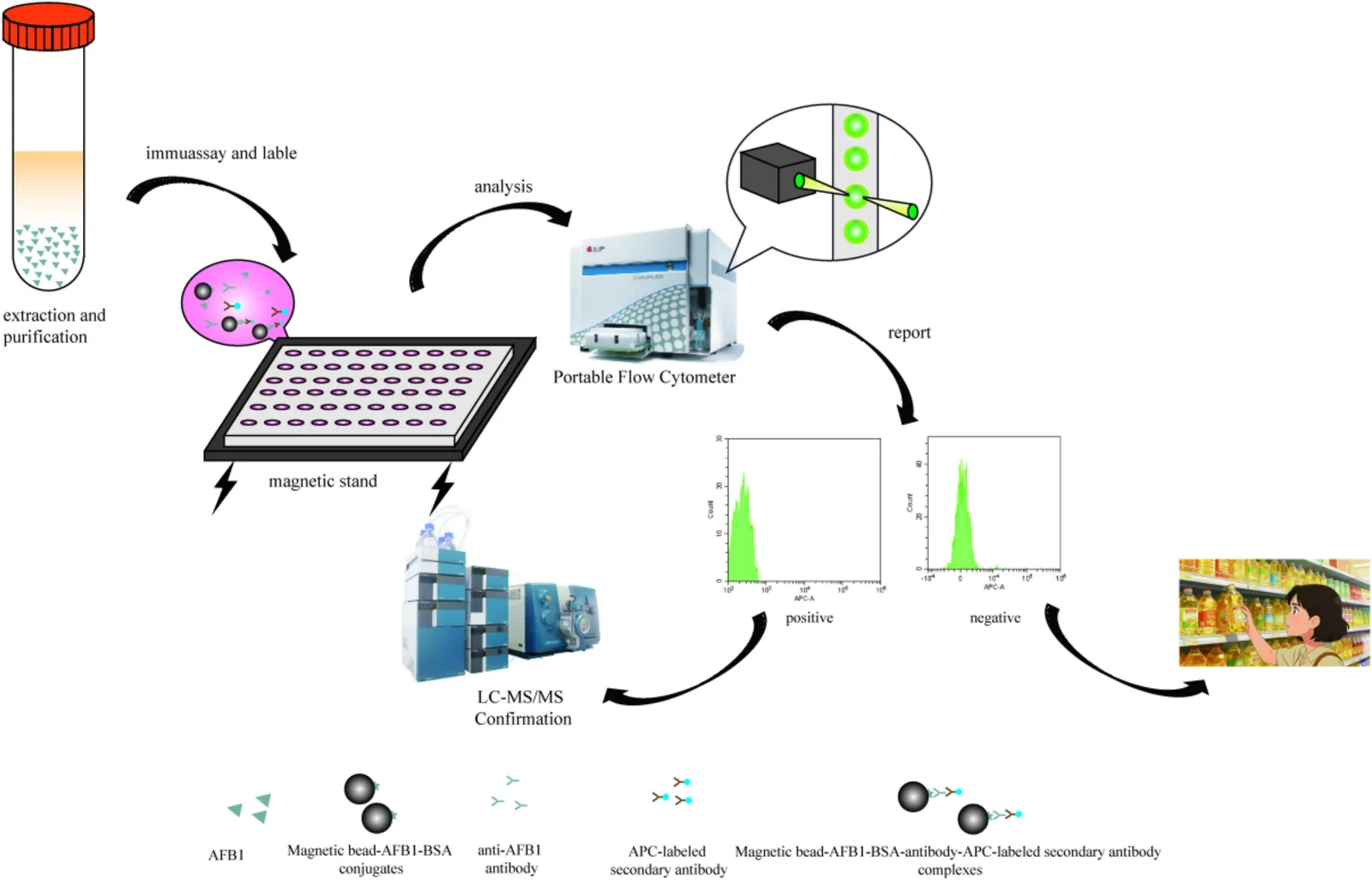
Schematic diagram of the method.

The working principle of the proposed system is illustrated in Fig. 1. First, a simple extraction and purification method is applied to the edible oil sample. The resulting extract is then added to a 96-well plate containing magnetic bead-AFB1-BSA conjugates. An appropriate amount of antibody is subsequently added to initiate a competitive reaction. After the reaction, magnetic bead-AFB1-BSA-antibody complexes are formed. The supernatant, which contains unbound antibody-AFB1 complexes and sample matrix, is removed using a magnetic stand. Next, a suitably concentrated APC-labeled secondary antibody is introduced for fluorescence labeling. Following this reaction, magnetic bead-AFB1-BSA-antibody-APC-labeled secondary antibody complexes were obtained. Finally, the fluorescence intensity of these complexes is measured using a flow cytometer, and the corresponding inhibition rate is calculated. The AFB1 content in the sample is determined from a concentration-inhibition standard curve. If a sample tests positive, the developed UHPLC-QqQ MS/MS method is employed to detect four aflatoxins (AFB1, AFB2, AFG1, AFG2) in the sample extract. This detection system design offers a dual advantage. On one hand, it utilizes the portability, high throughput, and high sensitivity of flow cytometry to screen out the majority of negative edible oil samples, thereby saving significant laboratory resources and manpower. On the other hand, for samples detected as positive, UHPLC-QqQ MS/MS is used for high-precision quantification or confirmation, effectively avoiding false-positive results.

#### 2.3.1 Preparation of magnetic bead-AFB1-BSA conjugates

Using a micropipette, 10 μL of streptavidin-modified magnetic microspheres was transferred into a 1.5 mL EP tube. Then, 1000 μL of PBS was added, followed by vortexing for 10 s. The tube was placed on a magnetic rack and allowed to stand for 3 min to facilitate adsorption. After adsorption, the supernatant was carefully removed with a micropipette. Next, 5 μL of biotin-modified AFB1 antigen and 995 μL of PBS were added to the tube. The mixture was incubated in a thermostatic mixer for 60 min (37 °C, 1000 rpm). After incubation, the tube was vortexed for 10 s and placed on the magnetic rack for 3 min. The supernatant was then carefully aspirated and discarded. The microspheres were washed once with 1000 μL of PBS following the same magnetic separation and supernatant removal steps. Subsequently, 1000 μL of 5% BSA blocking solution was added, and the tube was incubated in the thermostatic mixer for 120 min (37 °C, 1000 rpm). Following incubation, the tube was vortexed for 10 s and placed on the magnetic rack for 3 min. The supernatant was removed, and the microspheres were washed once again with 1000 μL of PBS using the same procedure. Finally, the microspheres were resuspended in 0.05% ProClin300 in PBS and stored at 4 °C.

#### 2.3.2 Sample preparation

Two grams of edible oil sample was weighed into a 15 mL centrifuge tube. Then, 100 mg of PSA and 5 mL of methanol were added. The mixture was vortexed for 5 min using a multi-tube vortex mixer and subsequently centrifuged at 10,000 rpm for 15 min. The resulting supernatant was collected for analysis by flow cytometry or UHPLC-QqQ MS/MS.

#### 2.3.3 MFC assay

In each well of a 96-well plate, 20 μL of the magnetic bead-AFB1-BSA conjugates was first added, followed by 10 μL of the sample extract or standard solution. Then, 10 μL of the diluted AFB1 antibody (1:100 dilution) and 60 μL of PBS were added. The plate was incubated in a thermomixer for 30 min (37 °C, 1000 rpm). After incubation, the plate was vortexed for 10 s and placed on a magnetic rack for 1 min to allow adsorption. The supernatant was removed, and 100 μL of PBS was added. The washing step (vortex, magnetic separation, and supernatant removal) was repeated once. Finally, 80 μL of PBST and 20 μL of the diluted APC-labeled secondary antibody (1:200 dilution) were added, and the plate was incubated again in the thermomixer for 30 min (30 °C, 1000 rpm). Following this incubation, the samples were analyzed using a flow cytometer.

#### 2.3.4 UHPLC-QqQ MS/MS assay

An appropriate volume of the sample extract was passed through a 0.22 μm filter membrane, and the filtrate was subjected to UHPLC-QqQ MS/MS analysis. Chromatographic separation was performed on a Waters BEH-C18 column (2.1 × 50 mm, 1.7 μm) maintained at 40 °C. The mobile phase consisted of purified water (mobile phase A) and acetonitrile (mobile phase B) with the following gradient program: 0–1 min, 10% B; 1–5 min, 10–95% B; 5–8 min, 95% B; 8–9 min, 95–10% B; 9–10 min, 10% B. The injection volume was 1 μL, and the flow rate was 0.4 mL/min. An ESI ion source was used, and the mass spectrometry was operated in positive ion mode with multiple reaction monitoring (MRM). The mass spectrometric parameters are listed in Table 1.

**Table 1:** MS/MS parameters for determination of 4 aflatoxins.

Analyte	Precursor ion (*m*/*z*)	Product ion (m/z)^a^	DP (V)	CE (eV)
AFB_1_	313.1	285.2 Q	170	31
		241.1C	170	50
AFB_2_	315.1	287.1 Q	150	34
		259.1C	150	41
AFG_1_	329.1	311.1 Q	135	30
		243.1C	190	38
AFG_2_	331.1	313.1 Q	185	34
		245.1C	170	42

a : Q, quantification ion; C, confirmation ion.

#### 2.3.5 Optimization strategy for the MFC immunoassay

A one-factor-at-a-time (OFAT) approach was employed to systematically optimize the key variables affecting the performance of the MFC assay. The following parameters were sequentially evaluated: (1) the preparation of magnetic bead-AFB1-BSA conjugates, including the number of washing cycles and magnetic adsorption time; (2) the amounts of key immunoreagents (biotin-modified AFB1 antigen, AFB1 polyclonal antibody, and APC-labeled fluorescent secondary antibody) and the concentration of the blocking agent (BSA); (3) the immunoreaction conditions (temperature and duration for the antigen-antibody reaction and the subsequent fluorescence labeling step); (4) the composition of the reaction medium; (5) the tolerance to organic solvents (methanol and acetonitrile); (6) the dosage of PSA sorbent for matrix cleanup; and (7) the cross-reactivity with other mycotoxins. For each optimization experiment, the performance was assessed based on the mean fluorescence intensity (MFI) or the signal-to-background ratio, and the condition yielding the optimal response was selected for subsequent experiments.

### 2.4 Method validation

A comprehensive and detailed validation protocol was conducted for both the MFC screening method and the confirmatory UHPLC-QqQ MS/MS method to evaluate their performance characteristics, including sensitivity, linearity, accuracy, precision, and specificity. The validation experiments were designed to comply with internationally accepted guidelines for bioanalytical method validation.

#### 2.4.1 Calibration, linearity, and sensitivity

For the MFC immunoassay, the calibration curve for AFB1 was established using a series of matrix-matched standard solutions in the range of 0.025 to 25 ng/g (0.025, 0.25, 0.625, 1.25, 6.25, 12.5, and 25 ng/g). The standard solutions were prepared in blank extracts of peanut oil, rapeseed oil, and olive oil. Each concentration level was analyzed in triplicate. The mean fluorescence intensity (MFI) data were fitted to a four-parameter logistic (4PL) nonlinear regression model using Origin 2024 software. The sensitivity was characterized by the half-maximal inhibitory concentration (IC50) and the IC10 value. The IC10, derived from the fitted curve, was defined as the practical limit of detection (LOD) for screening purposes.

For the UHPLC-QqQ MS/MS method, matrix-matched calibration curves were constructed for each aflatoxin (AFB1, AFB2, AFG1, AFG2) in each of the three oil matrices (peanut, rapeseed, olive). The calibration curves were established at seven concentration levels, specifically, 1.25, 2.5, 5, 10, 25, 50, and 125 ng/g for AFB_1_ and AFG_1_, and 0.5, 1, 2, 4, 10, 20, and 50 ng/g for AFB_2_ and AFG_2_. A minimum of six concentration levels were used for each curve, with each level injected in triplicate. The linearity was assessed by the correlation coefficient (R^2^) of the weighted (1/x) least-squares regression. The limit of detection (LOD) and limit of quantification (LOQ) for each analyte in each matrix were determined using serial dilutions prepared with the blank matrix extracts of peanut oil, rapeseed oil, and olive oil based on signal-to-noise ratios (S/N) of approximately 3.3 and 10, respectively. It should be noted that these LOD/LOQ values were derived from matrix-matched extract-based calibration standards (i.e., analytes spiked into post-extraction blank matrix extracts), and therefore reflect the instrument detection capability in the presence of matrix background. They do not account for potential analyte losses during the sample extraction and cleanup procedures, and should be regarded as matrix-matched instrument LOD/LOQ rather than full-method LOD/LOQ.

#### 2.4.2 Accuracy and precision

The accuracy (recovery) and precision (RSD) of both methods were evaluated through meticulously designed spike-and-recovery experiments.

Blank matrix preparation and verification: Authentic, uncontaminated samples of peanut oil, rapeseed oil, and olive oil were used as blank matrices. Prior to spiking, these oils were confirmed to be free of the target aflatoxins (AFB1 for MFC; AFB1, B2, G1, G2 for UHPLC-QqQ MS/MS) by the established UHPLC-QqQ MS/MS method, with all analyte concentrations below their respective limits of detection (LODs).

Spiking procedure: Working standard solutions of AFB1 (for MFC) or a mixture of the four aflatoxins (for UHPLC-QqQ MS/MS) were prepared in methanol. For each recovery test, a precise volume of the standard solution was spiked directly into 2.0 g of the blank oil matrix in a 15 mL centrifuge tube to achieve the target concentrations of 1, 2, 3 and 10 ng/g. The spiked samples were then vortexed thoroughly for 2 min to ensure homogeneous distribution of the analytes in the oil before proceeding with the sample preparation described in Section 2.3.2.

Definition of replicates and experimental design: For each combination of oil matrix (peanut, rapeseed, olive) and spiking level (1, 2, 3 and 10 ng/g), six independently prepared samples (*n* = 6) were analyzed. “Independently prepared” means that each replicate underwent the entire analytical process separately, starting from weighing 2.0 g of blank oil, spiking, extraction/cleanup with PSA, to final detection by MFC or UHPLC-QqQ MS/MS. This design assesses the overall method variability, including sample preparation and instrumental analysis.

Precision assessment: The six independently prepared replicates for each condition were analyzed within a single day to determine intra-day (repeatability) precision. To evaluate inter-day (intermediate) precision, the entire spike-and-recovery experiment (all three matrices at all four levels, *n* = 6 per condition) was repeated on six non-consecutive days over a two-week period.

#### 2.4.3 Specificity

The specificity of the MFC immunoassay was assessed by testing its inhibitory effect with other mycotoxins potentially found in edible oils. The tested compounds included AFB2, AFG1, AFG2, deoxynivalenol (DON), zearalenone (ZEA), ochratoxin A (OTA), fumonisin B1 (FB1), fumonisin B2 (FB2), and patulin (PAT). inhibitory effect was calculated using the formula: (MFIb-MFIs) / MFIb. A inhibitory effect value below 10% was considered negligible.

### 2.5 Data analysis

Flow cytometry data were processed using CytExpert software to obtain fluorescence intensity values, which were subsequently analyzed in Origin 2024 software to calculate inhibition rates, generate concentration–inhibition curves, and determine AFB1 concentrations. For UHPLC-QqQ MS/MS analysis, peak areas derived from Analyst software were further processed in Origin 2024 software to establish concentration–peak area calibration curves and quantify aflatoxin concentrations. All method validation data and graphical analyses were performed using Origin 2024 software, One-way analysis of variance (one-way ANOVA) combined with Tukey's post hoc test was used to compare the significant differences in fluorescence intensity (MFIt) under different optimization conditions (such as PSA concentration, antigen/antibody concentration, reaction temperature, etc.), with the significance level set at *p* < 0.05.

Unless otherwise stated, all other experiments were performed in triplicate (*n* = 3). Method validation experiments (accuracy and precision) were performed with six replicates (*n* = 6) at each spiked concentration level. Results are expressed as mean ± RSD (%).

## 3 Results and discussion

### 3.1 Optimization of MFC

A one-factor-at-a-time (OFAT) approach was adopted for condition optimization, as it provides a straightforward and practical strategy for identifying robust working conditions in immunoassay development without requiring complex statistical modeling. In the development of the MFC method, the difference between the mean fluorescence intensity of the sample (MFIs) and that of the blank (MFIb), denoted as MFIt, was used as the evaluation metric. Systematic optimization was performed for several key parameters, including the amounts of antigen, antibody, and fluorescent secondary antibody, as well as reaction temperature, reaction time, organic solvent content, and cross-reactivity.

#### 3.1.1 Preparation and optimization of Immunosensing elements

**Fig. 2:**
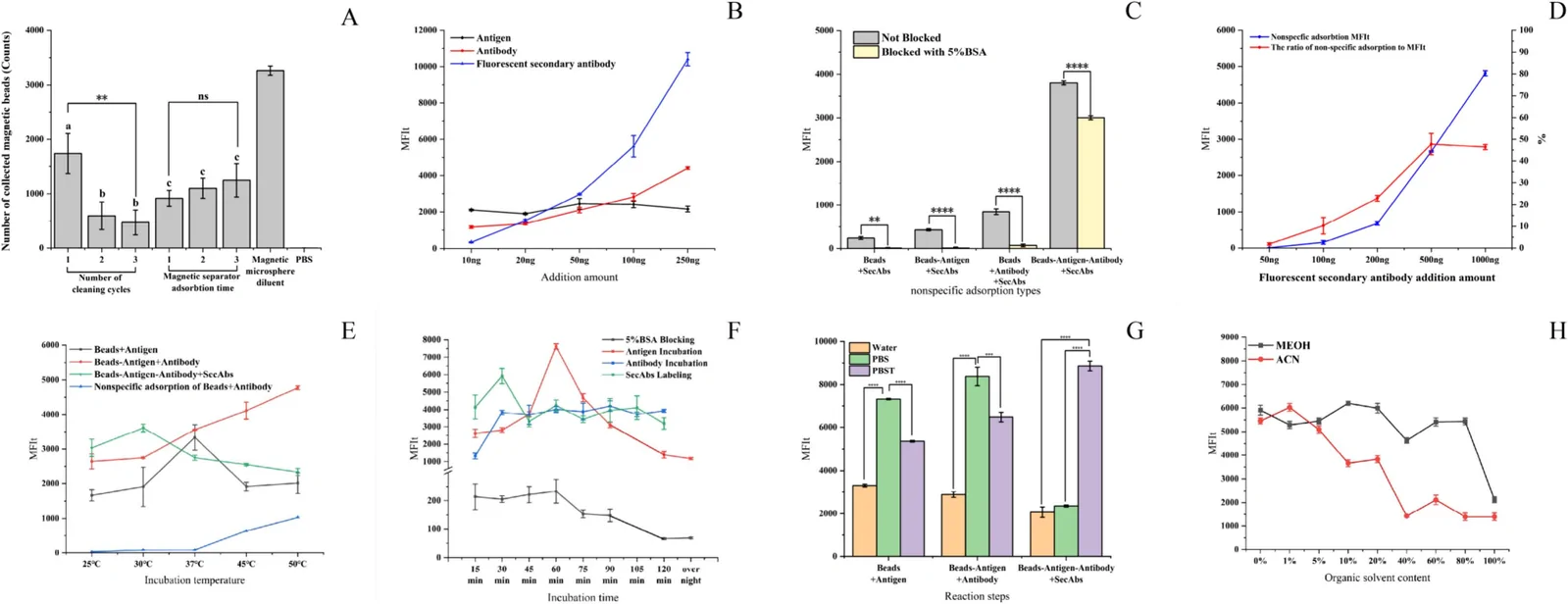
Optimization of conditions for the MFC method.(A. Comparison of data for the number of magnetic bead washing cycles and magnetic rack adsorption time. B. Comparison of different amounts of antigen, antibody, and fluorescent secondary antibody added. C. Comparison of the use of 5% BSA blocking in different incubation steps. D. Comparison of nonspecific adsorption and nonspecific adsorption rates for different amounts of fluorescent secondary antibody added. E. Comparison of different temperatures in different incubation steps. F. Comparison of reaction times in different steps. G. Comparison of reaction media in different steps. H. Comparison of different organic solvent concentrations. Data were expressed as mean ± SD (*n* = 3). Statistical differences among groups were evaluated by one-way ANOVA followed by Tukey's post hoc test using Origin 2024. *p < 0.05, **p < 0.01, ***p < 0.001, ****p < 0.0001; ns, not significant.) .

A magnetic rack enables rapid separation of magnetic beads from other components in solution via magnetic force, offering a more economical, convenient, and rapid alternative to ultrafiltration tubes. In the flow cytometry method for ochratoxin A (OTA) detection reported by Kong et al. (2017), three washing cycles and a magnetic adsorption time of 2 min were used, yet the effects of washing frequency and adsorption time on the measurable bead count were not thoroughly investigated. In this study, magnetic bead suspension diluted 5000-fold (5.44 × 10^3^ beads/mL) was subjected to varying numbers of washing cycles and different magnetic adsorption durations. The number of beads detectable within a defined region by flow cytometry within one minute was recorded. As shown in Fig. 2A, almost no particulate interference was observed in PBS. However, as the number of washing cycles increased, the number of detectable beads decreased significantly. After one, two, and three washing cycles, the bead counts were 53%, 18%, and 15% of that without washing, respectively (*p* < 0.01, one-way ANOVA). This marked decline contrasts with the study by Kong et al., who used carboxyl-modified beads, whereas the present study employed streptavidin-coated beads. The higher protein content on the surface of the beads used here may have affected their magnetic properties. However, as can be seen from Fig. 2A, the magnetic rack adsorption time showed no significant effect on the outcome (*p* > 0.05). Therefore, the optimal conditions were determined as a magnetic adsorption time of 1 min and one washing cycle.

This study compared the effects of varying amounts of antigen, antibody, and fluorescent secondary antibody added at 10, 20, 50, 100, and 250 ng. As shown in Fig. 2B, when the number of magnetic beads was 5.44 × 10^3^ and the antibody amount was 100 ng, no statistically significant difference was observed among the different antigen amounts (*p* > 0.05). However, when the antibody amount was increased to 250 ng, the highest MFIt value was achieved with an antigen addition of 50 ng (*p* < 0.01, one-way ANOVA). Further increase of the antigen amount to 100 ng and 250 ng led to a decrease in the mean fluorescence intensity. This may be attributed to the presence of multiple biotin modifications on the antigen surface; at excessively high concentrations, the antigen may bind to multiple magnetic beads, leading to aggregation and stacking. This phenomenon could hinder antibody binding and subsequently affect labeling by the fluorescent secondary antibody, thus reducing the MFIt value(Liu et al. (2018)). As illustrated in Fig. 2B, the MFIt value increased with higher antibody input, reaching its maximum at 250 ng (*p* < 0.01, one-way ANOVA). At this level, the ratio of antigen to antibody (50:250) was similar to that reported in previous studies (400:1500)(Liu et al. (2018)). Furthermore, the MFIt value increased significantly with the amount of fluorescent secondary antibody added, peaking at 250 ng (*p* < 0.01, one-way ANOVA). However, existing literature(Zhao et al. (2021)) suggested that excessive amounts of fluorescent secondary antibody may cause nonspecific fluorescence, which could compromise the sensitivity and accuracy of the detection method.

To identify the main causes of nonspecific MFIt, this study compared three potential sources of nonspecific MFIt. As shown in Fig. 2C, nonspecific adsorption of antibodies onto magnetic beads contributed the most to nonspecific MFIt, accounting for 22%, while the other two sources contributed only 6% and 11%, respectively. Furthermore, the effect of varying the amount of fluorescent secondary antibody on nonspecific MFIt was investigated. It was observed that nonspecific MFIt increased with higher amounts of fluorescent secondary antibody, reaching the highest levels at 500 ng and 1000 ng (*p* < 0.01, one-way ANOVA), where it accounted for over 46% of the total MFIt. In contrast, nonspecific MFIt remained below 23% at amounts of 100 ng, 200 ng, and 500 ng, as detailed in Fig. 2D. Considering the overall impact of nonspecific adsorption, the amount of fluorescent secondary antibody was ultimately optimized and set at 500 ng for subsequent experiments.

Numerous studies(Liu et al. (2021); Aqai, Peters, Gerssen, Haasnoot, and Nielen (2011); Vidal, Bertolín, Ezquerra, Hernández, and Castillo (2017)) have indicated that blocking with a BSA-containing solution can effectively reduce nonspecific fluorescence in magnetic bead–antigen conjugates. In this study, both bare magnetic beads and bead–antigen conjugates were incubated in a blocking buffer containing 5% BSA (4 °C, overnight), followed by addition of the fluorescent secondary antibody. As a result, the nonspecific adsorption, measured by MFIt, decreased significantly by 95% and 97%, respectively. Similarly, when the blocked beads were incubated with the primary antibody and then labeled with the fluorescent secondary antibody, the nonspecific MFIt signal was also markedly reduced by 92%. Detailed results are presented in Fig. 2C. The optimal conditions for the assay were finalized as follows: the antigen was used at 50 ng, blocking was performed overnight with 5% BSA solution, the primary antibody was added at 250 ng, and the fluorescent secondary antibody was used at 500 ng.

#### 3.1.2 Optimization of reaction conditions

To optimize the performance of the limited antigen, antibody, and fluorescent secondary antibody, this study systematically optimized the reaction temperature, reaction time, and reaction medium.

As shown in Fig. 2E, the reaction temperature showed specificity for the interaction between streptavidin-modified magnetic beads and biotin-modified antigen. The highest MFIt was observed at 37 °C, indicating the greatest coupling efficiency under this condition (*p* < 0.01, one-way ANOVA). At other temperatures tested, the MFIt values were similar to each other, reaching only 50–60% of that at 37 °C. This result is consistent with numerous ELISA methods utilizing streptavidin-biotin interactions, which also commonly employ 37 °C as the optimal reaction temperature(Urusov et al. (2015); Zhang et al. (2018); Liu, Nie, Zhao, Meng, and Wu (2015)). Therefore, this study further investigated the effect of reaction temperature on the interaction between the antibody and the bead-antigen conjugate. As shown in Fig. 2E, the amount of antibody coupled to the bead-antigen conjugate increased with higher temperatures. However, considering that nonspecific adsorption of the antibody to the beads had the most significant interference, the influence of temperature on nonspecific adsorption was also examined. It was found that nonspecific adsorption was relatively low at 25 °C, 30 °C, and 37 °C, but increased markedly at 45 °C and 50 °C (*p* < 0.01, one-way ANOVA), reaching 5 to 12 times the levels observed at lower temperatures (Fig. 2E). Based on these results, 37 °C was selected as the optimal reaction temperature for the antibody and bead-antigen conjugate. Additionally, Fig. 6 shows that the highest median fluorescence intensity (MFI) was achieved at 30 °C. The decrease in MFI at higher temperatures may be attributed to thermal quenching of the fluorescent dye.(Böcker et al. (2020); Li, Zhang, and Abbaspourrad (2022)).

Fig. 2F showed that the MFIt value reached its peak when the antigen was incubated with the magnetic beads for 60 min. Prolonging the reaction time led to a decrease in MFIt, with overnight incubation yielding only 15% of the MFIt obtained at 60 min. This reduction may have been due to bead aggregation induced by prolonged antigen exposure, which could encapsulate part of the antigen and prevent its subsequent binding with the antibody and fluorescent labeling(Liu et al. (2018)). Additionally, Fig. 2F indicated that blocking with 5% BSA solution for over 120 min effectively minimized nonspecific antibody adsorption to the beads, as reflected by a substantially lower MFIt value. Furthermore, the reaction time between the antibody and the bead-antigen complex, as well as the incubation time for the fluorescent secondary antibody, were optimized. As shown in Fig. 2F, antibody binding required more than 30 min to reach saturation and achieve the highest MFI, whereas incubation with the fluorescent secondary antibody for 30 min was sufficient. Extending the secondary antibody reaction beyond 30 min resulted in a decline in MFIt.

Fig. 2G shows that significant differences were observed among the three reaction media (purified water, PBS, and PBST) across the three-stage assay process. When PBS was used as the reaction medium, the highest MFIt values were achieved both during the antigen-bead conjugation step and the subsequent antibody binding step, exceeding those in the PBST group by 26% and 23%, respectively. PBST is commonly employed as a washing buffer in immunoassays such as ELISA, primarily to reduce nonspecific protein adsorption to the solid phase. Similarly, it may also interfere to some extent with specific immunoreactions(Kwon et al. (2009); Chen, Busnel, Peltre, Zhang, and Girault (2008); Reen (1994)). However, it was noteworthy that during the fluorescence labeling process, using PBST as the reaction medium significantly increased the MFI value, which was 3.7-fold and 4.3-fold higher than that obtained with PBS and purified water, respectively. Fig. 6 presents the flow cytometry scatter plots and histograms. The scatter plots showed that fluorescence labeling performed in purified water and PBS resulted in substantial aggregation of magnetic beads due to non-specific adsorption by the fluorescent secondary antibody, accounting for 57.75% and 30.90% of the total beads, respectively. In contrast, aggregated microspheres accounted for only 15.49% in samples labeled in PBST. The histograms indicated that the aggregated beads in purified water and PBS were heavily labeled with the fluorescent secondary antibody, with MFI values reaching 295,380 and 227,615, respectively, while the aggregated beads in PBST had a markedly lower MFI of 45,840. These aggregated beads were considered invalid in the detection system. These results demonstrated that in purified water and PBS, the fluorescent secondary antibody induces bead aggregation through non-specific adsorption and subsequently labels them, whereas PBST substantially suppresses such non-specific interactions. The primary reason was that while PBST provided a physiologically compatible pH and ionic strength, the Tween-20 it contained could continuously exert a steric hindrance effect, inhibiting both the aggregation of magnetic beads during the immunoassay and the nonspecific binding of impurity proteins from the sample matrix onto the solid-phase surface, thereby ensuring that the fluorescence signal truly originated from the target analyte. This allowed the magnetic beads to remain individually dispersed in the reaction medium, enabling the fluorescent secondary antibody to label each bead independently.

#### 3.1.3 Matrix effect management and Cleanup strategy

Edible oil, being a high-lipid matrix, was the sample type analyzed in this work. Consistent with literature reports, lipid-abundant matrices were known to pose potential interference in immunoassay systems(Qu et al. (2019); Meimaridou, Haasnoot, Shelver, Franek, and Nielen (2013); Wang et al. (2011)). The study investigated the matrix effect of peanut oil on the detection. It was found that direct testing of methanol extracts by flow cytometry caused interference, as shown in Fig. 8, where a distinct interfering signal appeared in the intensity range of 10,000–100,000 in the flow histogram. This interference was attributed to lipid components in the edible oil. The cleanup efficiency of primary secondary amine (PSA) sorbent, a key factor in removing interfering compounds such as fatty acids and pigments from the oil matrix, was systematically optimized. To systematically eliminate this interference, the cleanup procedure using Primary Secondary Amine (PSA) sorbent was optimized. The optimization was conducted using blank matrices of peanut oil. The key performance criterion was the complete removal of the non-specific high-intensity background signals in the MFC fluorescence histogram, ensuring a clean baseline for the specific detection channel. PSA dosage levels of 50 mg, 100 mg, and 150 mg were evaluated. The results were conclusive: Using 50 mg of PSA, the cleanup was insufficient. The MFC histograms consistently showed the persistent, problematic high-intensity background signals, indicating that matrix interferences remained. Both 100 mg and 150 mg of PSA were fully effective. The MFC analysis of extracts cleaned with these amounts resulted in histograms with a clean, low-background baseline, effectively eliminating the non-specific artifacts. There was no statistically significant difference (*p* > 0.05) in the efficacy of interference removal between the 100 mg and 150 mg doses. Therefore, 100 mg was selected as the optimal and standardized PSA dosage for all subsequent sample preparation. When the amount of PSA was increased from 50 mg to 100 mg, a greater number of adsorption sites effectively removed matrix interferences such as fatty acids and pigments from the oil, thereby eliminating the high-intensity nonspecific background signals in the flow cytometry plots. However, when the amount was further increased to 150 mg, the cleanup effect showed no significant improvement compared to 100 mg, indicating that 100 mg of PSA was sufficient to reach the adsorption saturation capacity for the given sample matrix and volume. Further increases in dosage provided no additional purification benefit. To extend the applicability of this cleanup method to more types of edible oils, matrix effects in 11 additional edible oils were examined. As shown in Fig. 4, the inhibition rates of 12 edible oils, including rapeseed oil and peanut oil, at an AFB1 concentration of 0.5 ng/g were comparable to those in buffer. This indicated that the cleanup method was suitable for MFC-based detection of AFB1 in a variety of edible oils.

#### 3.1.4 Evaluation of method tolerance and specificity

In immunoassays, when detecting molecules soluble in organic media, antibodies may begin to denature at certain high concentrations of organic solvents. This adversely affects the specific binding interaction between the antibody and antigen, leading to deviations in immunoassay results(An, Od, Ns, Av, and Bb (2024); Zhang et al. (2024)). As a hydrophobic compound, AFB1 requires organic solvents to facilitate dissolution; thus, the content of organic solvent is a critical factor to consider. In this study, two organic solvents commonly used for mycotoxin extraction, methanol and acetonitrile, were evaluated at concentrations of 0%, 1%, 5%, 10%, 20%, 40%, 60%, 80%, and 100%. As shown in Fig. 2H, methanol concentrations above 80% significantly impaired antibody-antigen recognition, whereas acetonitrile concentrations exceeding 5% markedly affected this specific interaction. This was likely due to the stronger hydrophobicity of acetonitrile, which at high levels can alter the hydrophobic/hydrophilic balance on the antibody surface, thereby interfering with specific binding(Lou, Zhu, Luo, Zhang, and Long (2017)). Furthermore, flow cytometry scatter plots (Fig. 7) revealed a sharp decline in the detected target magnetic beads when methanol concentration reached 100%, and only a small fraction of target beads were detectable at 60% acetonitrile. This may be attributed to structural degradation of the magnetic beads after exposure to high concentrations of organic solvents, leading to loss of spherical integrity and subsequent detection failure.

**Fig. 3:**
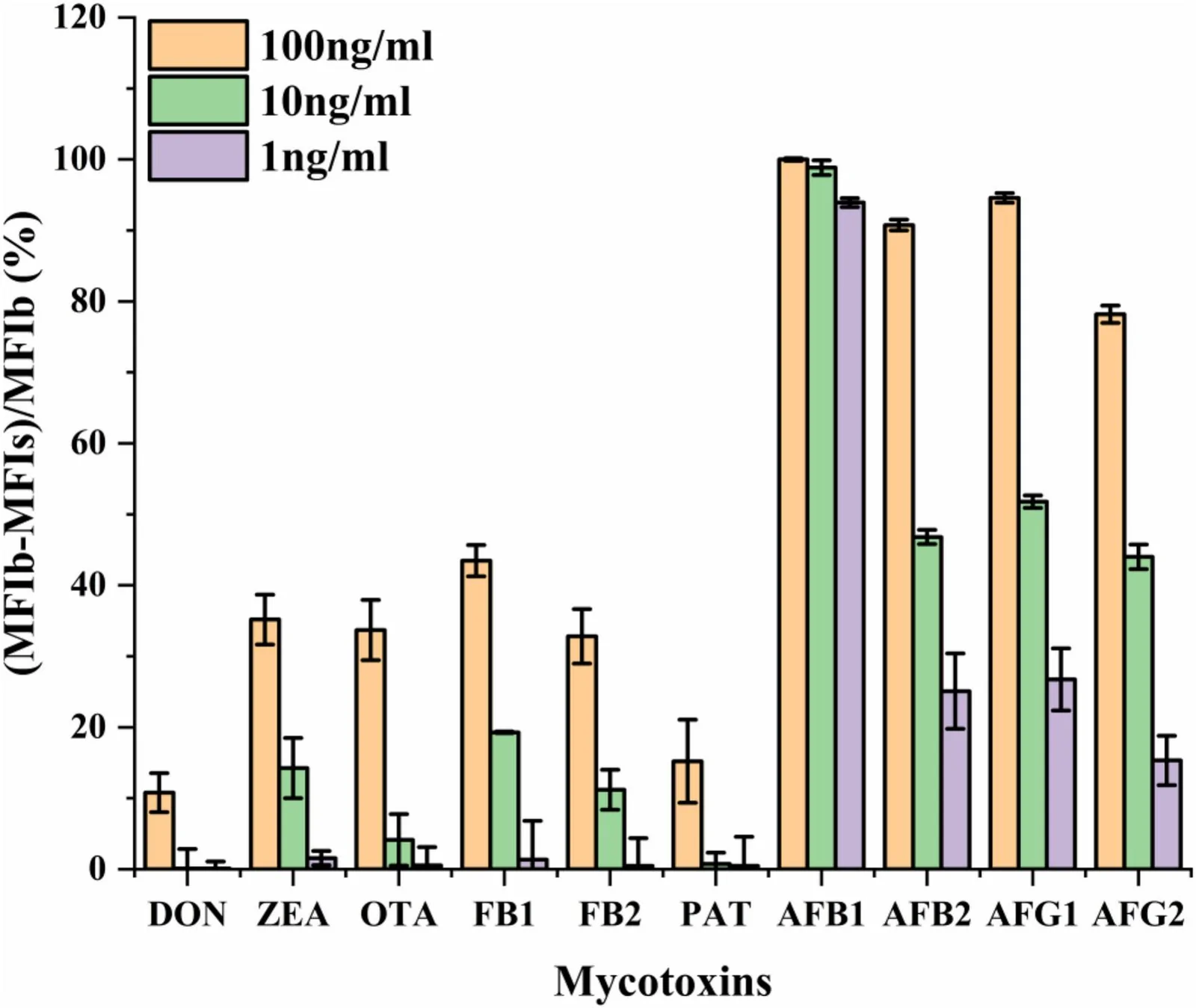
Cross-reactivity of the method with other potential mycotoxins.

Given that other mycotoxins may also be present in edible oils(Junsai et al. (2021)), such as deoxynivalenol (DON), zearalenone (ZEN), ochratoxin A (OTA), fumonisins B1 and B2 (FB1 and FB2), patulin (PAT), as well as the aflatoxin congeners AFB2, AFG1, and AFG2, potential interference with the proposed method should be taken into consideration. Therefore, we spiked three concentration levels (100, 10, and 1 ng/mL) of these mycotoxins to evaluate their inhibitory effects on the present method. The inhibition rate was expressed as (MFIb-MFIs) / MFIb, and the results are shown in Fig. 3. At 100 ng/mL, all tested mycotoxins exhibited varying degrees of inhibition. The homologous aflatoxins AFB2, AFG1, and AFG2 showed inhibition rates of 72% - 87%, while the inhibition rates of other mycotoxins ranged from 10% to 40%. When the concentration decreased to 10 ng/mL, the inhibition rates of DON and PAT dropped sharply to below 1%. ZEN, OTA, FB1, and FB2 exhibited inhibition rates of 4% - 18%, whereas the three homologous aflatoxins still maintained inhibition rates of 40% - 48%. At 1 ng/mL, the inhibition rates of OTA and FB2 also fell below 1%, while ZEN and FB1 remained at 4% - 7%. The three homologous aflatoxins continued to show inhibition rates of 14% - 25%. The primary reason for this pattern was that the method employs a polyclonal antibody, which may detect compounds structurally similar to AFB1. The subtle differences in the chemical structures of AFB2, AFG1, and AFG2, such as the saturation state of the dihydrofuran ring or variations in the cyclopentenone ring, significantly reduced their affinity for the antibody's binding domain. This design was intentional to avoid false-negative results, considering that the method was intended for rapid screening, with positive samples requiring further confirmation by mass spectrometry. Accordingly, an UHPLC-QqQ MS/MS method capable of simultaneously detecting the above ten mycotoxins was also developed in this study. In this study, an inhibition rate below 10% at low concentration levels is considered acceptable. Although the three homologous aflatoxins still exhibited inhibition rates above 10% even at low concentrations, these aflatoxins often co-occur and are regulated by legislation. Therefore, the detection of any aflatoxin should be further confirmed by the UHPLC-QqQ MS/MS method.

**Fig. 4:**
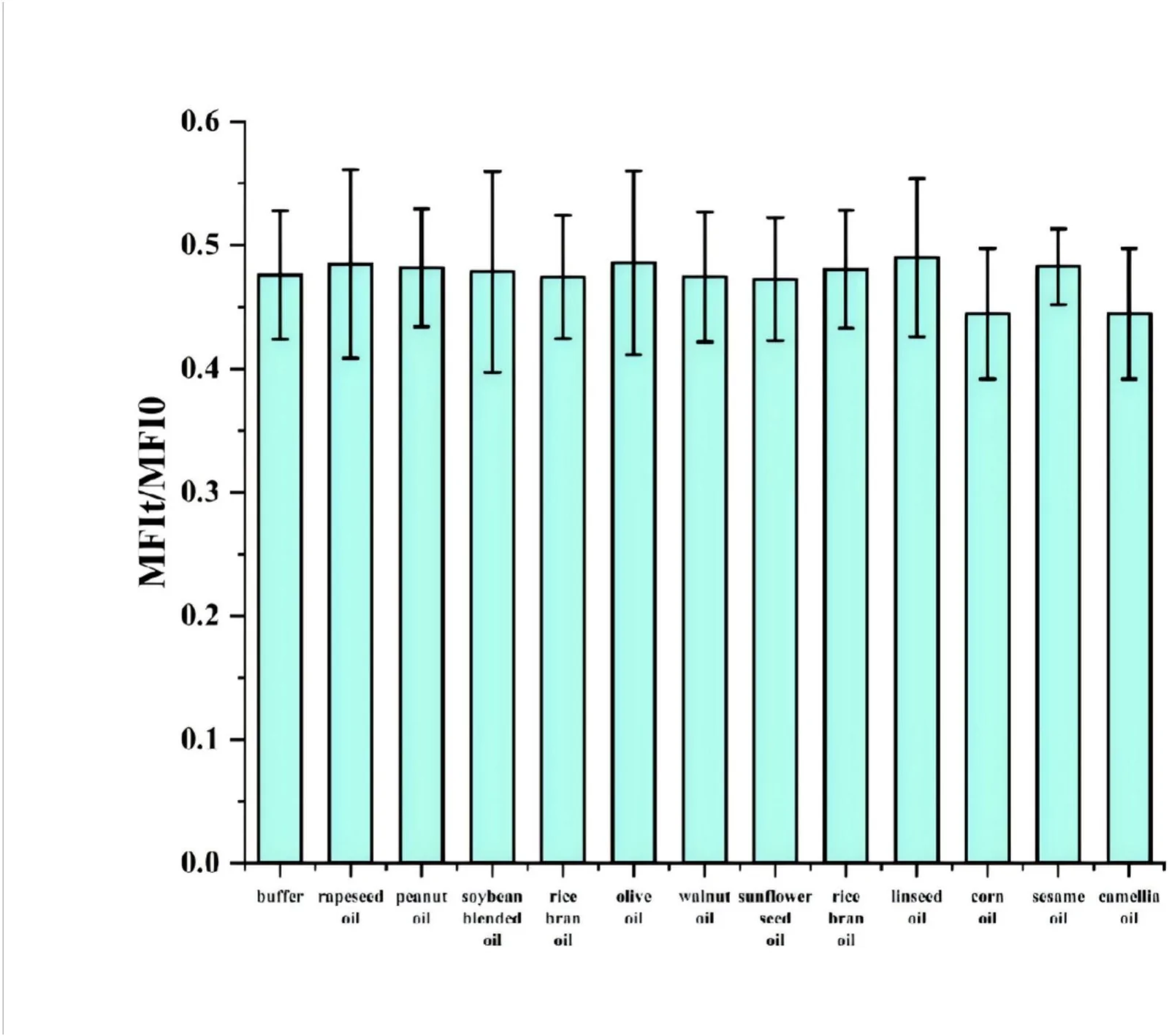
Inhibition rate performance of the developed method across different matrices.

**Fig. 5:**
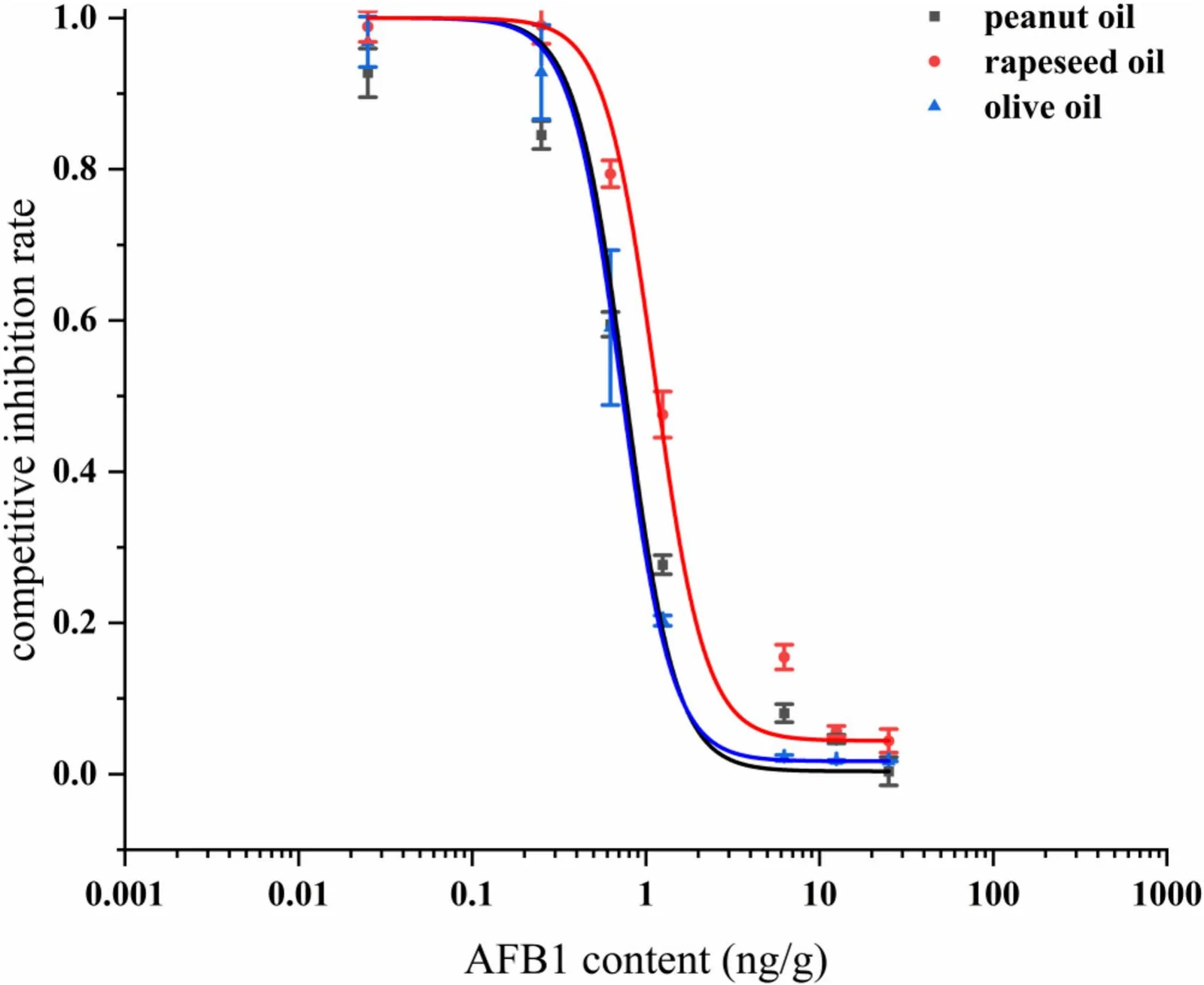
Competitive inhibition curve of the MFC method.

**Fig. 6:**
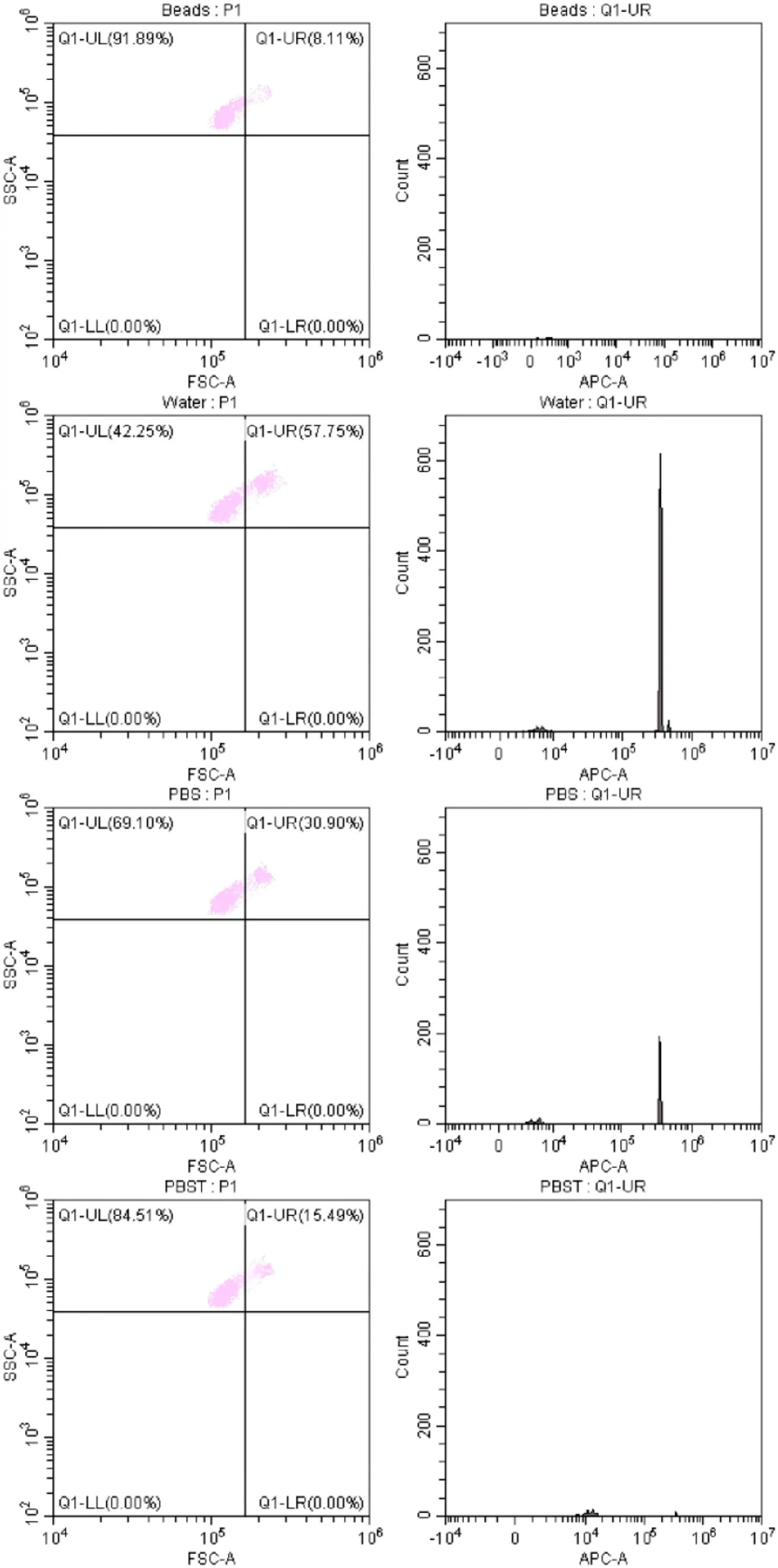
Flow cytometry plots showing the results after reaction in different incubation media.

**Fig. 7:**
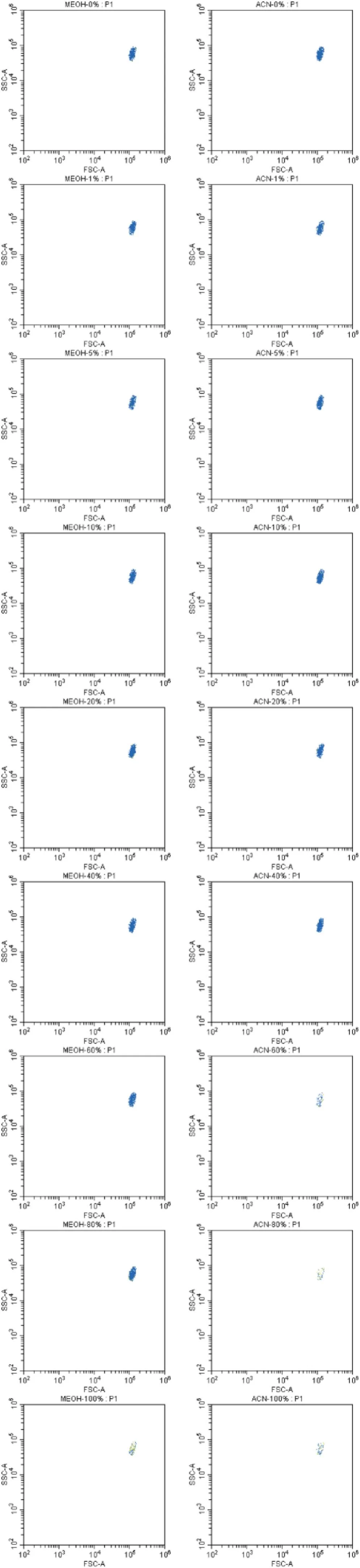
Influence of Varying Organic Solvent Content on Flow Cytometric Analysis.

**Fig. 8:**
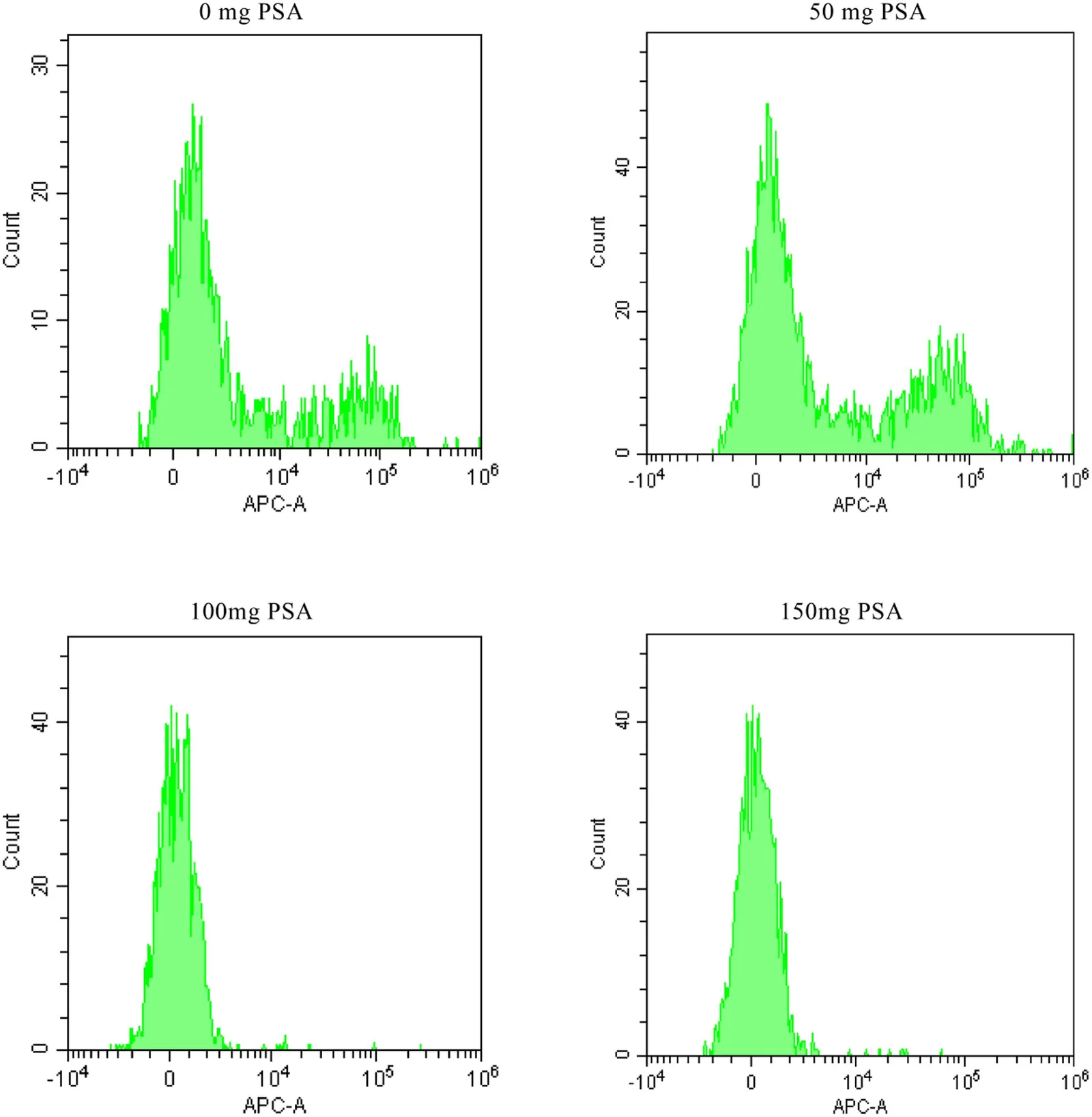
Effect of different PSA concentrations on flow cytometric analysis.

The observed cross-reactivity pattern can be rationalized from a structural perspective. AFB1, AFB2, AFG1, and AFG2 share a common difuranocoumarin chromophore, which constitutes the primary epitope recognized by the polyclonal antibody. The structural differences among these analogues, such as the saturation of the dihydrofuran ring (AFB2 vs. AFB1) or the substitution of the cyclopentenone ring with a lactone moiety (AFG1/AFG2 vs. AFB1/AFB2), introduce subtle steric and electronic variations that modulate antibody-binding affinity. AFB2 and AFG1, which retain the difuranocoumarin core with minimal structural perturbation, exhibited the highest cross-reactivity (72–87% at 100 ng/mL), consistent with their structural similarity to the immunogen. In contrast, non-aflatoxin mycotoxins such as OTA, ZEN, and DON, which lack this core scaffold, showed negligible interference (<10% at low concentrations). This selective cross-reactivity profile confirms that the polyclonal antibody maintains broad recognition of aflatoxin analogues while effectively discriminating against structurally unrelated mycotoxins, a design feature that ensures comprehensive screening of the aflatoxin family without significant false-positive risk from co-occurring contaminants.

### 3.2 Method validation

A comprehensive validation of both the established MFC screening method and the confirmatory UHPLC-QqQ MS/MS method was performed to evaluate their accuracy, sensitivity, precision, and applicability. The validation was conducted using blank matrices of three representative edible oils: peanut oil, rapeseed oil, and olive oil.

For the MFC method, the sensitivity for AFB₁ detection in different matrices was characterized by the IC10 and IC50 values derived from the nonlinear calibration curve (Fig. 5). The IC10 value was used as the practical limit of detection (LOD) for screening purposes. The method demonstrated a broad dynamic range from 0.025 ng/g to 25 ng/g for AFB₁ across all tested oil matrices (Table 2). For the UHPLC-QqQ MS/MS method, the limits of detection (LOD) and quantification (LOQ) for each aflatoxin (AFB₁, AFB₂, AFG₁, AFG₂) were determined in each oil matrix using serial dilutions prepared with the blank extracts of peanut oil, rapeseed oil, and olive oil, defined as signal-to-noise ratios of approximately 3.3 and 10, respectively. The method exhibited excellent sensitivity suitable for regulatory compliance (Table 3). The linearity was assessed using matrix-matched calibration curves, showing good linear relationships in the ranges of 1.25–125 ng/g for AFB₁ and AFG₁, and 0.50–50 ng/g for AFB₂ and AFG₂, with correlation coefficients (R^2^) greater than 0.99 for all analytes in all three oil matrices.

**Table 2:** Method validation parameters for MFC.

Analyte	Linear range (ng/g)	Matrix type	Linear formula	R^2^	IC10⁎ (ng/g)	Mean recovery (%, *n* = 6)					Inter-day precision (%, n = 6)		
AFB_1_	0.025–25	peanut oil	y = 0.04412 + 0.89766 / (1 + (x / 0.7629) ^ 2.0246)	0.99657	0.26	Spiked Concentration	1 ng/g	98.05	RSD (%)	5.41	Spiked Concentration	1 ng/g	7.18
							2 ng/g	101.47		6.35		2 ng/g	5.67
							3 ng/g	102.73		4.82		3 ng/g	5.32
							10 ng/g	99.24		4.18		10 ng/g	9.54
AFB_1_	0.025–25	rapeseed oil	y = 0.03797 + 0.96854 / (1 + (x / 1.32175) ^ 1.61613)	0.99725	0.34	Spiked Concentration	1 ng/g	91.02	RSD (%)	7.46	Spiked Concentration	1 ng/g	9.00
							2 ng/g	97.84		6.26		2 ng/g	7.94
							3 ng/g	94.83		5.40		3 ng/g	7.25
							10 ng/g	94.97		4.03		10 ng/g	7.18
AFB_1_	0.025–25	olive oil	y = 0.0169 + 0.97401 / (1 + (x / 0.7036) ^ 2.50785)	0.99982	0.29	Spiked Concentration	1 ng/g	90.51	RSD (%)	4.66	Spiked Concentration	1 ng/g	4.76
							2 ng/g	98.66		9.25		2 ng/g	7.45
							3 ng/g	96.08		6.76		3 ng/g	6.51
							10 ng/g	96.43		4.64		10 ng/g	6.49

⁎ : equivalent to the Limit of Detection (LOD).

**Table 3:** Method validation parameters for UHPLC-QqQ MS/MS.

Matrix type	Analyte	LOD/LOQ (ng/g)	Linear range (ng/g)	Linear formula	R^2^	Spiked Concentration (ng/g)	Mean recovery (%, n = 6)	RSD (%)	Inter-day precision (%, n = 6)
peanut oil	AFB_1_	0.40/1.25	1.25–125	y = 154,866 x + 131,374	0.9995	1	90.57	2.18	2.73
						2	103.26	5.46	5.90
						3	94.88	3.71	4.36
						10	95.52	3.56	6.24
	AFB_2_	0.20/0.50	0.50–50	y = 155,832 x + 74,826	0.9974	1	99.41	1.34	1.90
						2	107.12	5.01	5.58
						3	91.35	4.28	4.75
						10	103.65	6.11	6.98
	AFG_1_	0.40/1.25	1.25–125	y = 154,913 x + 149,928	0.9992	1	105.78	1.09	1.73
						2	97.03	4.52	6.11
						3	101.69	5.83	6.05
						10	99.52	5.43	6.49
	AFG_2_	0.20/0.50	0.50–50	y = 156,266 x + 74,943	0.9984	1	93.20	2.97	3.53
						2	88.99	3.19	3.80
						3	106.55	1.67	2.31
						10	103.17	5.02	6.37
rapeseed oil	AFB_1_	0.40/1.25	1.25–125	y = 158,087 x + 109,456	0.9996	1	95.90	3.29	8.51
						2	96.40	7.93	9.28
						3	102.54	4.39	7.65
						10	101.72	3.95	3.68
	AFB_2_	0.20/0.50	0.50–50	y = 155,984 x + 55,412	0.9981	1	94.66	6.32	5.52
						2	98.18	8.15	7.65
						3	102.06	6.48	7.58
						10	103.11	3.37	5.94
	AFG_1_	0.40/1.25	1.25–125	y = 154,508 x + 152,111	0.9991	1	101.60	3.64	5.39
						2	99.79	9.94	7.76
						3	104.00	5.11	9.30
						10	103.95	3.89	5.44
	AFG_2_	0.20/0.50	0.50–50	y = 155,042 x + 58,522	0.9993	1	95.52	6.29	6.42
						2	93.43	4.76	10.58
						3	94.15	7.43	10.03
						10	99.57	4.48	6.89
olive oil	AFB_1_	0.40/1.25	1.25–125	y = 157,708 x + 120,948	0.9995	1	94.35	6.85	7.46
						2	96.18	4.12	6.70
						3	101.75	4.85	9.87
						10	101.82	3.25	6.82
	AFB_2_	0.20/0.50	0.50–50	y = 154,264 x + 56,462	0.9991	1	95.31	8.24	10.43
						2	94.84	8.85	8.11
						3	100.60	7.51	7.00
						10	102.56	5.60	5.36
	AFG_1_	0.40/1.25	1.25–125	y = 153,449 x + 167,048	0.9989	1	93.54	4.41	7.57
						2	96.04	5.49	5.72
						3	100.42	7.96	8.33
						10	99.56	4.06	5.41
	AFG_2_	0.20/0.50	0.50–50	y = 152,909 x + 56,559	0.9992	1	100.66	6.79	5.96
						2	98.80	5.23	7.25
						3	95.95	10.19	8.29
						10	103.38	4.42	6.68

The accuracy of both methods was evaluated through recovery experiments. Blank oil matrices (peanut, rapeseed, olive) were spiked with target analytes at four concentration levels (1, 2, 3 and 10 ng/g), representing approximately 50%, 100%, 150% and 500% of the EU regulatory limit for AFB₁. The inclusion of this broader concentration range was intended to evaluate method performance not only near the regulatory threshold but also at significantly elevated contamination levels, thereby demonstrating the robustness and applicability of the method across different risk scenarios. Six replicates were analyzed for each level and each matrix. As summarized in Table 2, the MFC method for AFB₁ showed mean recoveries ranging from 90.51% to 102.73% across the three oil matrices, with intra-day precision (expressed as relative standard deviation, RSD) between 4.03% and 9.25%. The inter-day precision, evaluated over six consecutive days, yielded RSDs from 4.76% to 9.54%. The UHPLC-QqQ MS/MS method demonstrated equally satisfactory performance (Table 3). For the four aflatoxins in the three oil matrices, the mean recoveries at the four spiked levels ranged from 88.99% to 107.12%. The intra-day precision RSDs were between 1.09% and 10.19%, while the inter-day precision RSDs ranged from 1.73% to 10.58%. All recovery and precision results met the accepted criteria for bioanalytical method validation, confirming the high reliability of both methods.

The consistently high recoveries (90.51–102.73%) and acceptable precision (RSD < 10%) observed across three high-lipid edible oil matrices are attributable to the effectiveness of the PSA cleanup procedure in removing matrix-interfering components. Edible oils, particularly peanut and rapeseed oils, contain abundant free fatty acids, phospholipids, and pigments that can non-specifically bind to immunoreagents or interfere with fluorescence detection, leading to signal suppression or elevation. The primary amine groups of PSA sorbent act as weak anion exchangers, effectively retaining acidic interfering compounds such as free fatty acids through ionic interactions, while its secondary amine moieties provide additional retention mechanisms for polar pigments and phenolic compounds. This dual retention mechanism ensures thorough matrix cleanup without significant analyte loss. The achieved recovery performance compares favorably with recent immunoassay studies in lipid-rich matrices: Qu et al. (2019) reported recoveries of 82–115% for aflatoxins in milk using a magnetic bead-based flow cytometry immunoassay after extensive sample preparation, while Meimaridou et al. (2013) observed recoveries of 78–104% for AFB1 in maize using ELISA with immunoaffinity column cleanup. The present method achieves comparable or superior recovery performance with a significantly simplified extraction and cleanup workflow, demonstrating that the PSA-based approach provides effective matrix mitigation for reliable quantitative screening in challenging lipid matrices.

To evaluate the robustness of the proposed MFC method, the effects of minor deliberate variations in critical analytical parameters were investigated. The influence of PSA sorbent amount (95, 100, and 105 mg) on extraction efficiency was assessed at the 2 ng/g spiking level, with recovery RSD values below 10%, indicating satisfactory tolerance to variations in cleanup sorbent dosage. The effects of incubation time (29, 30, and 31 min) and temperature (primary antibody: 36, 37, and 38 °C; secondary antibody: 29, 30, and 31 °C) on immunoreaction performance were evaluated by monitoring the mean fluorescence intensity (MFI). Under all tested variations, the MFI RSD values remained below 10%, demonstrating that the assay is robust against minor fluctuations in incubation duration and temperature. These results confirm that the proposed method exhibits satisfactory robustness within the tested parameter ranges, supporting its reliability for routine application under typical laboratory or field conditions.

The storage stability of the key immunoreagents was evaluated to assess their long-term performance under recommended storage conditions. Magnetic microspheres stored at 4 °C and antigen/antibody reagents stored at −20 °C for up to 12 months were tested for their immunoreactivity using the established MFC assay. At the 2 ng/g spiking level (*n* = 3), the recoveries were 87.76%, 92.03%, and 89.19%, with a relative standard deviation (RSD) of 1.98%, indicating that the original reagents retained satisfactory activity after one year of storage under the specified conditions. However, it should be noted that once prepared, the magnetic bead–AFB1-BSA conjugates, diluted primary antibody working solution (1:100), and APC-labeled secondary antibody working solution (1:200) exhibited significant performance deterioration when stored at 4 °C for more than 3 months. Therefore, to ensure reliable and reproducible assay results, it is recommended that working solutions be freshly prepared or used within 3 months of preparation when stored at 4 °C.

### 3.3 Analysis of real samples

The practicality and reliability of the established dual-mode screening strategy were evaluated by analyzing 39 real edible oil samples. This set comprised 29 batches of commercially available edible oils (including peanut, soybean, rapeseed, and blended oils) purchased from local markets, and 10 batches of artificially contaminated peanut oil samples prepared in the laboratory to simulate naturally moldy conditions. All samples were first analyzed using the portable MFC method for rapid, on-site quantitative screening of AFB1. The screening criterion was set at the IC10 value (0.26–0.34 ng/g depending on the matrix, as per Table 2). Samples yielding signals below the IC10 were judged as negative and excluded from further confirmatory analysis. Samples with signals at or above the IC10 were marked as presumptive positive and proceeded to the second stage. In the second stage, these presumptive positive samples underwent confirmatory analysis using the UHPLC-QqQ MS/MS method. This step provided precise quantification of AFB1, as well as the specific content of AFB2, AFG1, and AFG2, ensuring accurate results for regulatory compliance. The results are summarized in Table 4. For all 29 commercially available oil samples, the MFC screening results were below the IC10, correctly identifying them as negative. This was conclusively confirmed by the UHPLC-QqQ MS/MS analysis, which showed that the concentrations of all four aflatoxins were below their respective limits of detection (LODs). This demonstrates the high efficiency of the MFC screening step, which can reliably exclude a large number of negative samples, thereby saving significant laboratory time and resources. The absence of detectable aflatoxin contamination in all 29 commercially available edible oil samples is consistent with recent contamination surveys in China, which indicate that regulatory enforcement and improved storage practices have substantially reduced aflatoxin levels in commercially processed oils. Ji, Jiang, Zhang, Hou, and Sun (2022) surveyed 63 plant oil products from retail markets in China and reported that only 39.7% of samples contained detectable aflatoxins, with AFB1 levels predominantly below 2 ng/g. Similarly, a nationwide monitoring program conducted by the China National Food Safety Risk Assessment Center (CFSA) in 2021–2022 found that over 95% of commercial edible oil samples complied with the national AFB1 limit of 20 μg/kg, with peanut oil exhibiting the highest compliance rate (98.3%). The negative results in the present study further corroborate the effectiveness of current regulatory measures, including mandatory aflatoxin testing at oil processing facilities and strict enforcement of maximum residue limits. Nevertheless, the presence of aflatoxin in 10 artificially contaminated samples at levels exceeding the EU limit (2 ng/g) in 7 out of 10 cases underscores the continued risk of contamination under improper storage conditions and highlights the importance of maintaining robust surveillance systems, particularly for small-scale producers and informal market channels where monitoring may be less stringent.

**Table 4:** Sample analysis results by MFC and UHPLC-QqQ MS/MS.

Types of edible oils	Lot Number	AFB_1_(ng/g)		AFB_2_(ng/g)		AFG_1_(ng/g)		AFG_2_ (ng/g)	
		MFC	UHPLC-QqQ MS/MS	MFC	UHPLC-QqQ MS/MS	MFC	UHPLC-QqQ MS/MS	MFC	UHPLC-QqQ MS/MS
rapeseed oil	20,240,908	<IC_10_	<LOD	/	<LOD	/	<LOD	/	<LOD
	20,240,306	<IC_10_	<LOD	/	<LOD	/	<LOD	/	<LOD
	20,250,101	<IC_10_	<LOD	/	<LOD	/	<LOD	/	<LOD
	20,240,907	<IC_10_	<LOD	/	<LOD	/	<LOD	/	<LOD
	20,240,520	<IC_10_	<LOD	/	<LOD	/	<LOD	/	<LOD
	20,241,117	<IC_10_	<LOD	/	<LOD	/	<LOD	/	<LOD
	20,231,022	<IC_10_	<LOD	/	<LOD	/	<LOD	/	<LOD
	20,240,509	<IC_10_	<LOD	/	<LOD	/	<LOD	/	<LOD
peanut oil	20,241,218	<IC_10_	<LOD	/	<LOD	/	<LOD	/	<LOD
	20,250,120	<IC_10_	<LOD	/	<LOD	/	<LOD	/	<LOD
	20,250,108	<IC_10_	<LOD	/	<LOD	/	<LOD	/	<LOD
	20,250,111	<IC_10_	<LOD	/	<LOD	/	<LOD	/	<LOD
	20,240,918	<IC_10_	<LOD	/	<LOD	/	<LOD	/	<LOD
	20,241,219	<IC_10_	<LOD	/	<LOD	/	<LOD	/	<LOD
	20,241,030	<IC_10_	<LOD	/	<LOD	/	<LOD	/	<LOD
	20,240,826	<IC_10_	<LOD	/	<LOD	/	<LOD	/	<LOD
	20,231,119	<IC_10_	<LOD	/	<LOD	/	<LOD	/	<LOD
	20,241,212	<IC_10_	<LOD	/	<LOD	/	<LOD	/	<LOD
	20,241,215	<IC_10_	<LOD	/	<LOD	/	<LOD	/	<LOD
	Quality control sample 1	20.06	12.49	/	7.42	/	12.84	/	11.43
	Quality control sample 2	26.37	12.51	/	6.54	/	11.23	/	10.31
	Quality control sample 3	22.08	10.78	/	6.29	/	11.74	/	11.91
homemade peanut oil	2,025,041,601	3.71	3.65	/	<LOD	/	<LOD	/	<LOD
	2,025,041,602	10.89	9.36	/	<LOD	/	<LOD	/	<LOD
	2,025,041,603	1.50	1.26	/	<LOD	/	<LOD	/	<LOD
	2,025,041,604	5.18	4.87	/	<LOD	/	<LOD	/	<LOD
	2,025,041,605	0.86	1.07	/	<LOD	/	<LOD	/	<LOD
	2,025,041,606	4.59	4.02	/	<LOD	/	<LOD	/	<LOD
	2,025,041,607	3.06	3.13	/	<LOD	/	<LOD	/	<LOD
	2,025,041,608	1.08	1.15	/	<LOD	/	<LOD	/	<LOD
	2,025,041,609	8.82	8.61	/	<LOD	/	<LOD	/	<LOD
	2,025,041,610	2.05	2.04	/	<LOD	/	<LOD	/	<LOD
spiked homemade peanut oil	2,025,041,602	11.75	9.13	/	0.94	/	0.98	/	0.96
	2,025,041,605	7.78	1.03	/	5.21	/	4.97	/	5.34
soybean blended oil	20,241,024	<IC_10_	<LOD	/	<LOD	/	<LOD	/	<LOD
rice bran oil	20,241,228	<IC_10_	<LOD	/	<LOD	/	<LOD	/	<LOD
olive oil	20,241,220	<IC_10_	<LOD	/	<LOD	/	<LOD	/	<LOD
walnut oil	20,241,125	<IC_10_	<LOD	/	<LOD	/	<LOD	/	<LOD
sunflower seed oil	20,240,424	<IC_10_	<LOD	/	<LOD	/	<LOD	/	<LOD
rice oil	20,241,127	<IC_10_	<LOD	/	<LOD	/	<LOD	/	<LOD
linseed oil	20,240,909	<IC_10_	<LOD	/	<LOD	/	<LOD	/	<LOD
corn oil	20,240,114	<IC_10_	<LOD	/	<LOD	/	<LOD	/	<LOD
sesame oil	20,250,107	<IC_10_	<LOD	/	<LOD	/	<LOD	/	<LOD
camellia oil	20,241,122	<IC_10_	<LOD	/	<LOD	/	<LOD	/	<LOD

/: not detected.

Quality control samples (*n* = 3; AFB_1_ levels: 10.78, 12.49, and 12.51 ng/g by UHPLC-QqQ MS/MS) were included solely for method performance monitoring and were excluded from the prevalence and regulatory compliance analyses.

For the 10 artificially contaminated peanut oil samples, the MFC method successfully identified all as presumptive positive. The confirmatory UHPLC-QqQ MS/MS analysis verified the presence of AFB1 in all these samples, with concentrations ranging from 1.07 ng/g to 9.36 ng/g (Table 4). Notably, the AFB1 concentrations determined by the MFC method showed a high degree of agreement with those obtained by the UHPLC-QqQ MS/MS method, demonstrating the accurate quantitative capability of the MFC screening assay. The concentrations of AFB2, AFG1, and AFG2 in these samples were all below their LODs as determined by UHPLC-QqQ MS/MS. This result reflects the naturally occurring AFB1-dominant contamination pattern in moldy peanut oils, rather than any limitation of the UHPLC-QqQ MS/MS method in detecting other aflatoxins. It is also noted that the MFC method, by design, is not highly specific to AFB1 alone; its polyclonal antibody exhibits cross-reactivity with AFB2, AFG1, and AFG2 (as demonstrated in Section 3.1.4), which is an intentional feature to minimize false-negative screening results. The absence of detectable co-occurring aflatoxins in these samples is therefore attributable to the natural contamination profile, not to the analytical specificity of either method.

The absence of co-occurring aflatoxins in these samples is fully consistent with previously reported contamination patterns in edible oils. In a survey of 63 plant oil products from retail markets in China, Ji et al. (2022) detected aflatoxins in 25 positive samples, with a 76% co-occurrence rate. However, AFB1 was consistently the dominant congener, accounting for 12.1%–98.2% of total aflatoxins and up to 68% in highly contaminated oils. The authors attributed this predominance to the higher lipophilicity of AFB1, which facilitates its partitioning into the oil phase, whereas AFG2, the most hydrophilic of the four analogues, exhibits lower extractability in fat-soluble solvents. Additionally, *Aspergillus flavus*, which primarily produces B-group aflatoxins (AFB1 and AFB2), is more prevalent in oil crop contamination than *A. parasiticus*, which produces all four types. This AFB1-dominant pattern was similarly observed by Idris, Mariod, Elnour, and Mohamed (2010) in Sudanese edible oils, where AFB1 was the predominant contaminant in all positive samples. Thus, the contamination profile observed in our samples aligns with the predominant mode of aflatoxin contamination reported in the global literature.

To systematically evaluate the performance of the MFC method under co-occurrence scenarios, we extended our analysis to include three commercial peanut oil quality control reference materials and conducted two controlled spiking experiments (Table 4). In the three quality control samples (QC1, QC2, QC3), the MFC yielded apparent AFB1 concentrations of 20.06–26.37 ng/g, substantially higher than the UHPLC-QqQ MS/MS reference values of 10.78–12.51 ng/g. This positive bias reflects the intentional design of the polyclonal antibody, which maintains cross-reactivity with AFB2, AFG1, and AFG2 to minimize the risk of false negatives. In a further experiment, we spiked batch 2,025,041,605 (baseline AFB1 approximately 1 ng/g) with elevated levels of AFB2, AFG1, and AFG2 (approximately 5 ng/g each). Under these conditions, the MFC yielded an apparent AFB1 concentration of 7.78 ng/g, overestimating the UHPLC-measured AFB1 (1.03 ng/g) by approximately 655%, confirming significant cross-reactivity when co-contaminants are abundant.

However, as the literature confirms, the vast majority of contaminated edible oil samples feature AFB1 as the overwhelmingly dominant toxin. Under such realistic low-level co-occurrence conditions, which we simulated by adding small amounts of AFB2 (0.94 ng/g), AFG1 (0.98 ng/g), and AFG2 (0.96 ng/g) to batch 2,025,041,602, the MFC result (11.75 ng/g) remained in strong agreement with the UHPLC value (9.13 ng/g). This demonstrates that at the co-contaminant levels typically encountered in practice, the MFC method provides reliable AFB1 quantification. Importantly, even in high co-contamination scenarios where the MFC cannot precisely resolve individual aflatoxin species, no positive sample is missed; all trigger presumptive positive signals and are subsequently subjected to UHPLC-QqQ MS/MS for precise multi-analyte quantification. This two-tier design, MFC for inclusive screening, UHPLC-QqQ MS/MS for definitive confirmation, ensures robustness to variable co-occurrence patterns while optimizing analytical resources.

In conclusion, the analysis of real samples validated the entire workflow of the dual-mode strategy. The MFC method served as a reliable and rapid screening tool, while the UHPLC-QqQ MS/MS method provided definitive confirmation and precise quantification. This strategy is well-suited for high-throughput market surveillance, enabling efficient on-site screening followed by accurate laboratory confirmation only for suspect samples.

### 3.4 Comparison with mainstream rapid detection methods

To objectively highlight the advantages of the proposed dual-mode screening system, a direct performance comparison was conducted with two widely used commercial rapid detection kits: lateral flow immunoassay (LFIA) strips and enzyme-linked immunosorbent assay (ELISA). The key operational and performance parameters, obtained from testing these commercially purchased kits alongside our methods, are summarized in Table 5.

**Table 5:** Performance comparison of the developed methods with commercial rapid test kits for AFB1 detection in edible oils.

Feature / Method	Commercial LFIA Strips (Tested in this study)	Commercial ELISA Kit (Tested in this study)	Portable MFC Method (This work)	Dual-Mode System (MFC + UHPLC-QqQ MS/MS) (This work)
Detection Principle	Immunochromatography	Immunoassay + colorimetry	Immunoassay + magnetic separation + fluorescence	Immunoassay (screening) + Chromatography-MS (confirmation)
Result Type	Qualitative / Semi-quantitative (visual)	Quantitative	Quantitative	Quantitative (screening & confirmation)
Sensitivity (LOD for AFB1)	5 ng/g (visual)	0.1 ng/g	0.26–0.34 ng/g (IC₁₀)	0.26–0.34 ng/g (MFC screening); <0.5 ng/g (UHPLC-QqQ MS/MS confirmation)
Total Assay Time	∼15 min/sample	>2 h	Batch of 96 samples in ∼180 min (∼1 min/sample reading speed; ∼32 samples/h throughput)	∼1 min/sample (screening) + 15–20 min/sample (confirmation for positives only)
Throughput Potential	Low (single test per strip)	Medium (batch of 96 samples)	High (continuous flow, ∼60 samples/h)	Very High (efficient screening drastically reduces confirmatory load)
Multi-analyte Capacity	Single (AFB1)	Single (AFB1)	Single (AFB1)	Yes (AFB1, B2, G1, G2 via UHPLC-QqQ MS/MS)
Instrument Dependency	None (visual) or reader	Plate washer & reader	Portable analyzer (field-deployable)	Portable analyzer + Lab-based UHPLC-QqQ MS/MS
Ease of On-site Use	Excellent (minimal training)	Poor (requires lab setting)	Good (simple protocol, portable)	Good (on-site screening step)
Approx. Cost per Test	Low	Medium	Medium	Medium-High (overall cost-effective by reducing confirmatory tests)
Regulatory Action Data	No (Presumptive)	Usually no (Presumptive)	No (Presumptive)	Yes (Definitive confirmation by UHPLC-QqQ MS/MS)
Main Advantage	Ultimate simplicity, very low cost	High sensitivity, good for lab batch processing	Rapid, quantitative, portable, high throughput	Optimal balance: High-efficiency on-site screening + Definitive laboratory confirmation
Main Limitation	Qualitative/low sensitivity, not confirmatory	Time-consuming, lab-bound, complex steps	Not a confirmatory method	Requires access to two platforms

Note: The performance data for the commercial LFIA strips and ELISA kit were obtained from experimental tests conducted in this study using the purchased kits. The data for the MFC and Dual-Mode system are from this study.

As shown in Table 5, the developed portable MFC method demonstrates clear practical benefits for on-site screening. Compared to the tested LFIA strips, which provided only qualitative or semi-quantitative visual results, the MFC method delivers fully quantitative data with superior sensitivity (IC10 of 0.26–0.34 ng/g vs. the visual LOD of 5 ng/g observed for the strips). The MFC assay also offers significant time savings. Owing to its 96-well plate format, all sample preparation and immunoassay steps (extraction, competitive reaction, magnetic separation/washing, secondary antibody incubation) are performed in parallel for the entire batch. The flow cytometric reading step proceeds at approximately 1 min per sample, yielding a batch throughput of ∼32 samples/h with only ∼30 min of hands-on operator time. By comparison, the LFIA strip test requires approximately 15 min per individual test with no parallelization, and the ELISA kit requires a total assay time exceeding 2 h. While the tested ELISA kit showed excellent sensitivity, it involved a more complex and time-consuming procedure (total assay time > 2 h) requiring laboratory equipment, making it unsuitable for rapid field deployment. In contrast, the MFC protocol, with its simplified magnetic separation and rapid fluorescence detection, offers a better balance of speed, ease of use, and quantitative capability at the point of need.

The core innovation of this work lies in the strategic integration of the MFC screening with confirmatory UHPLC-QqQ MS/MS into a coherent workflow. This dual-mode system addresses a key limitation of standalone rapid tests: the inability to provide regulatory-grade confirmatory data. Our approach efficiently leverages the strengths of both techniques. The portable MFC device enables rapid, quantitative on-site screening to filter out the majority of negative samples. Only the limited number of presumptive positive samples are then subjected to definitive, multi-analyte confirmation by UHPLC-QqQ MS/MS. This strategy significantly optimizes resource allocation in surveillance programs by reserving sophisticated laboratory analysis only for samples that truly warrant it, achieving an optimal balance of screening efficiency, operational practicality, and analytical certainty.

To further contextualize the analytical performance of the proposed system, Table 6 compares it with previously published MFC-based methods for mycotoxin detection. To the best of our knowledge, all existing MFC-based mycotoxin assays have been performed on conventional large benchtop flow cytometers under controlled laboratory conditions. The present study represents the first report of a compact, field-deployable MFC platform for aflatoxin detection in edible oils. As shown in Table 6, the proposed method achieved comparable or superior sensitivity relative to existing MFC approaches, while uniquely combining rapid sample preparation (<15 min), portability (instrument weight < 15 kg), and suitability for on-site screening in complex oil matrices.

**Table 6:** Comparison of the proposed method with previously published flow cytometry-based mycotoxin detection methods.

Method	Target	Matrix	Instrument	LOD / IC₁₀	IC₅₀	Analysis time	Quant.	Portable / Field- deployable	Ref.
MFC + UHPLC-QqQ MS/MS (this work)	AFB1	Edible oils	CytoFLEX (compact benchtop, <15 kg)	0.26–0.34 ng/g	0.76 ng/g	∼1 min/ sample; ∼32 samples/ h (96-well)	Yes	Yes	This study
CBA (magnetic microspheres)	OTA	Malt	Conventional benchtop FC	0.025 μg/kg	NR	∼2 h	Yes	No	Kong et al., 2017
FCIA (UCNMMs)	AFB1	NR	Conventional benchtop FC	NR	NR	NR	Yes	No	Zhang et al., 2017
Suspension array (Luminex)	AFB1	Corn, peanut	Conventional benchtop FC	NR	NR	NR	Yes	No	Wang et al., 2013
Quadplex FCIA	AFM1	Milk	Conventional benchtop FC	0.045 μg/L	NR	>1 h	Yes	No	Qu et al., 2019

NR: not reported.

In addition to MFC-based platforms, several recently developed rapid detection methods (2023–2025) warrant comparison to further evaluate the competitiveness of the proposed system. Surface-enhanced Raman scattering (SERS)-based aptasensors have achieved LODs as low as 0.039 ng/mL for AFB1, with recoveries of 92.77–110.13% in corn flour and oil matrices (Guo et al. (2024)); however, these methods require sophisticated nanoparticle substrates and benchtop Raman instrumentation, limiting their field deployability. Electrochemical immunosensors based on site-directed antibody conjugation strategies have reported LODs as low as 0.042 pg/mL for AFB1 in wheat and rice bran oil (Hao et al. (2025)); while offering improved portability through screen-printed electrodes, their reproducibility remains constrained by electrode-to-electrode variability, and most reported applications have been validated only in limited real-sample matrices. Click reaction-mediated fluorescent immunosensors using Cu-MOF nanoparticles have demonstrated LODs of 0.48 pg/mL with 670-fold sensitivity enhancement over conventional ELISA (Hong et al. (2024)); however, their quantitative accuracy relies on precise control of click reaction conditions and automated instrumentation, which may not be readily adaptable to resource-limited field settings. In comparison, the proposed MFC method achieves an IC10 of 0.26–0.34 ng/g in real edible oil matrices with fully quantitative output, high-throughput capacity (∼32 samples/h), and instrument portability (<15 kg), representing a more practical balance of sensitivity, throughput, and field deployability for large-scale regulatory screening applications.

### 3.5 Implications for regulatory compliance and practical monitoring

The dual-mode screening strategy established in this study provides a solution for the market surveillance of aflatoxins in edible oils that combines both high efficiency and high reliability. The portable MFC method serves as the initial screening tool, capable of efficiently identifying presumptively contaminated samples in batch mode (∼32 samples/h, based on a 96-well plate format with a flow cytometric reading speed of ∼1 min/sample). This allows for the exclusion of over 90% of negative samples in large-scale monitoring programs, as demonstrated in this study where none of the commercially purchased edible oils tested positive for any aflatoxin. This significantly reduced the burden on confirmatory instrumentation and the overall monitoring cost.

The contamination levels detected in the laboratory-prepared peanut oil samples (1.07–9.36 ng/g; Table 4) were interpreted against current regulatory maximum limits to illustrate the practical significance of the dual-mode system. Commission Regulation (EU) 2023/915 establishes maximum levels of 2 μg/kg for AFB1 alone and 4 μg/kg for the sum of AFB1 + B2 + G1 + G2 in vegetable oils intended for direct human consumption (European Commission. (2023)). China's GB 2761–2017 sets a limit of 20 μg/kg for AFB1 in peanut oil (National Health and Family Planning Commission of the People's Republic of China, and China Food and Drug Administration. (2017)). Of the 10 contaminated samples tested, 3 (1.07, 1.15, and 1.26 ng/g) fell below the EU AFB1 limit of 2 ng/g, while 7 (2.04, 3.13, 3.65, 4.02, 4.87, 8.61, and 9.36 ng/g) exceeded it. Notably, no sample exceeded the Chinese national limit of 20 ng/g. These findings confirm that the EU limit is substantially more stringent than the Chinese standard. The MFC method's low IC₁₀ screening level (0.26–0.34 ng/g) comfortably covers both regulatory requirements, ensuring that even samples marginally exceeding the EU limit can be reliably flagged for confirmatory analysis.

The co-occurrence profile of aflatoxins in our samples further informs the regulatory interpretation. In all 10 contaminated samples analyzed, only AFB1 was detected at concentrations above the LOD, with AFB2, AFG1, and AFG2 falling below their respective limits of detection. It is important to clarify that this observation reflects the naturally occurring AFB1-dominant contamination pattern in edible oils, as extensively documented in the literature, rather than serving as evidence of high analytical specificity of the MFC assay for AFB1. As demonstrated in the cross-reactivity experiments (Section 3.1.4), the MFC immunoassay responds to other aflatoxin analogues (AFB2, AFG1, AFG2) due to the nature of the polyclonal antibody, which was intentionally designed to avoid false negatives. The MFC method therefore functions as an inclusive screen for aflatoxins, with the UHPLC-QqQ MS/MS second stage providing the definitive species-specific identification. This observation aligns with the predominant contamination pattern documented in the literature. Ji et al. (2022) reported that AFB1 accounted for 12.1%–98.2% of total aflatoxins across 25 positive plant oil samples from Chinese retail markets, reaching up to 68% in highly contaminated oils. The authors attributed this to the higher lipophilicity of AFB1, which facilitates its partitioning into the oil phase during extraction, whereas the more hydrophilic AFG2 exhibits lower extractability in fat-soluble solvents. Similarly, Idris et al. (2010) found AFB1 to be the predominant contaminant in all positive Sudanese edible oil samples. Given this consistent AFB1-dominant pattern across geographically diverse studies, the MFC method's design, optimized for quantitative AFB1 screening while leveraging cross-reactivity to flag co-occurrence cases for confirmatory testing, is well suited to regulatory surveillance needs. The confirmatory UHPLC-QqQ MS/MS method simultaneously quantifies all four aflatoxins, directly addressing both the EU requirement for total aflatoxin assessment and the Chinese requirement for individual AFB1 quantification.

For all samples flagged as presumptive positive by the MFC screen, confirmatory analysis is performed using the UHPLC-QqQ MS/MS method. This method not only accurately quantifies AFB1 but also simultaneously detects AFB2, AFG1, and AFG2. This design is crucial for addressing the risk of co-occurrence contamination by multiple aflatoxins in real samples, as different toxins have varying toxicological equivalence and regulatory requirements. As the Table 4 demonstrates, the UHPLC-QqQ MS/MS method provides precise quantification of all four aflatoxins in quality control and spiked samples, while the MFC method reliably flags all positive cases regardless of the co-occurrence pattern. In summary, this dual-mode system achieves precise and efficient management of food safety risks under resource constraints by utilizing MFC for high-throughput cost savings and UHPLC-QqQ MS/MS to provide confirmed, comprehensive toxin data suitable for direct regulatory decision-making.

To further evaluate the practical feasibility and throughput capacity of the proposed method under field-relevant conditions, a large-scale on-site screening simulation was conducted at a local grain and oil market. A total of 87 edible oil samples were collected and analyzed on-site using the portable MFC screening mode. The entire sample preparation, MFC measurement, and data processing for all 87 samples were completed within 4 h, and no sample exceeded the IC10 threshold, indicating that all samples complied with the regulatory limit for AFB1. In contrast, analysis of the same sample set using conventional laboratory-based methods (HPLC or UHPLC-QqQ MS/MS method) would require a minimum of five working days due to instrument throughput limitations and sample queue constraints. This result demonstrates that the proposed method can support high-throughput, on-site screening in real-world regulatory monitoring scenarios, significantly reducing the turnaround time from sample collection to result delivery.

### 3.6 Limitations and future perspectives

This study has several limitations that warrant consideration. First, although the method was validated across three representative oil matrices (peanut, rapeseed, and olive oil), the total number of real samples analyzed (39 batches plus 87 batches in the field simulation) was relatively limited and primarily sourced from a single geographic region, which may not fully represent the diversity of contamination patterns across different production regions and oil varieties. Second, the portable MFC instrument used in this study, while compact (<15 kg), still requires a stable power supply and trained operators, which may pose challenges in some resource-limited field settings. Third, the present study was conducted as a single-laboratory validation, and inter-laboratory reproducibility has not yet been assessed. Finally, certain specialty oil matrices with strong inherent coloration or complex compositions (e.g., dark sesame oil, perilla oil) were not evaluated in this study and may require matrix-specific optimization.

Future work will focus on several directions to further enhance the practical utility of this platform. First, the development of multiplex MFC assays capable of simultaneously screening multiple mycotoxins (e.g., AFB1, OTA, DON, and ZEN) in a single workflow would significantly broaden the method's applicability across diverse food matrices. Second, integration of the sample extraction and MFC detection into a semi-automated or cartridge-based system could further reduce operator dependency and analysis time, enabling use by non-specialist personnel in field or small-scale production settings. Third, the combination of the MFC platform with signal amplification strategies, such as enzyme-mediated fluorescence enhancement or nanomaterial-based labels, may further lower the detection limit to address increasingly stringent regulatory requirements. Fourth, multicenter collaborative studies involving laboratories across different regions are planned to validate the method's reproducibility and transferability. Finally, extension of this dual-mode strategy to other high-risk food matrices, such as animal feeds, nuts, and spices, represents a promising direction for broader food safety applications.

## 4 Conclusion

In this study, a dual-mode screening strategy combining a portable MFC-based immunoassay with confirmatory UHPLC-QqQ MS/MS was developed and validated for the rapid, accurate detection of aflatoxins in edible oils. The MFC method, utilizing a simplified PSA-based sample cleanup and magnetic bead-based competitive immunoassay, achieved an IC10 of 0.26–0.34 ng/g and an IC50 of 0.76 ng/g for AFB1 in edible oil matrices, with a broad dynamic range (0.025–25 ng/g) and high throughput (∼32 samples/h). Comprehensive method validation across three representative oil matrices (peanut, rapeseed, and olive oil) confirmed the reliability of both methods, with mean recoveries of 90.51%–102.73% (MFC) and 88.99%–107.12% (UHPLC-QqQ MS/MS), and precision (RSD) values below 10.58%. Analysis of 126 real-world samples (39 laboratory-screened samples and 87 field-simulated batches) demonstrated the practical feasibility of the strategy, with the MFC method correctly identifying all negative samples within 4 h, reducing the confirmatory workload by over 90%. To the best of our knowledge, this represents the first report of a compact, field-deployable MFC platform for aflatoxin detection in edible oils. The proposed dual-mode system provides an effective and practical solution for large-scale regulatory screening of aflatoxins in edible oils, offering a balance of sensitivity, throughput, and on-site applicability that addresses current gaps in food safety surveillance.

## CRediT authorship contribution statement

**Guangyao Ying:** Writing – original draft, Methodology, Formal analysis, Data curation, Conceptualization. **Tingting Wang:** Writing – review & editing, Writing – original draft, Validation, Investigation, Formal analysis, Data curation. **Kunlun Li:** Resources, Methodology. **Zheng Lu:** Validation, Methodology, Conceptualization. **Yuxin Wang:** Visualization, Formal analysis. **Gangjian Lin:** Resources. **Jun Li:** Writing – review & editing, Validation, Supervision. **Huili Xia:** Writing – review & editing, Validation, Formal analysis. **Liang Hong:** Writing – review & editing, Supervision, Funding acquisition.

## Declaration of competing interest

The authors declare that they have no known competing financial interests or personal relationships that could have appeared to influence the work reported in this paper.

## Data Availability

Data will be made available on request.
